# Flavonoids: A Review of Antibacterial Activity Against Gram-Negative Bacteria

**DOI:** 10.1155/ijm/9961121

**Published:** 2025-09-15

**Authors:** Joice Barbosa do Nascimento, José Galberto Martins da Costa

**Affiliations:** ^1^Programa de Pós-Graduação em Química Biológica, Departamento de Química Biológica, Universidade Regional do Cariri, Crato, Ceará, Brazil; ^2^Laboratório de Pesquisa de Produtos Naturais, Departamento de Química Biológica, Universidade Regional do Cariri, Crato, Ceará, Brazil

**Keywords:** antibacterial effects, flavonoids isolated, Gram-negative, natural products

## Abstract

The rise in bacterial resistance to conventional antibiotics has led to the search for alternative antimicrobial agents, such as flavonoids, which are widely distributed in plants and known for their antibacterial properties. This review examines the antibacterial activity of flavonoids against Gram-negative bacteria and identifies gaps in current knowledge. The analysis of available data revealed that several flavonoids, such as liricidin-7-*O*-hexoside, licoflavone C, and dorsmanins C and F, demonstrated remarkable antibacterial potential. It was also observed that flavonoids exhibit multifactorial antibacterial activity, interfering with various cellular bacterial processes such as maintaining cell membrane integrity, energy metabolism, and protein synthesis. However, despite the growing number of studies indicating the promising potential of these compounds against bacterial pathogens, many of these studies mainly focus on in vivo evaluations, limited to verifying the presence or absence of antibacterial activity, without an in-depth investigation of the mechanisms of action or clinical efficacy of flavonoids. Additionally, most research has been conducted on a limited number of bacterial species, such as *Escherichia coli*, *Pseudomonas aeruginosa*, and *Klebsiella pneumoniae*. Furthermore, few studies have explored the effects of pure flavonoids, as most investigations have centered on extracts or flavonoid-rich fractions. There is a significant gap in research regarding the antibacterial activity of isolated flavonoids against Gram-negative bacteria. The review calls for further studies on pure flavonoids, focusing on understanding their mechanisms of action, interactions with other therapies, pharmacokinetics, toxicity, and clinical effectiveness to develop more targeted treatments for multidrug-resistant bacterial infections.

## 1. Introduction

**Figure 1:**
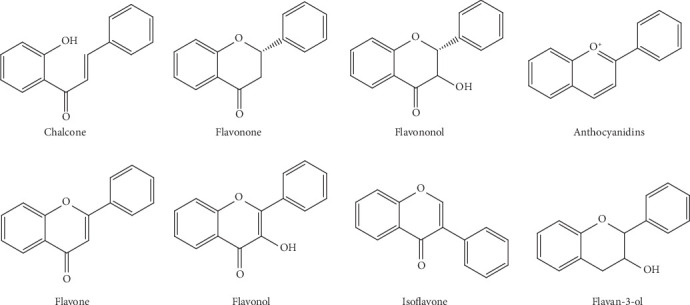
Flavonoid classes.

Flavonoids constitute one of the major classes of secondary metabolites in plant species, comprising more than 10,000 substances [1]. This group of polyphenolic compounds presents a benzo-*γ*-pyrone structure and is synthesized through various metabolic pathways, including the shikimate pathway, the phenylpropanoid pathway, and the flavonoid biosynthesis pathway [2]. Based on their chemical structure (C6-C3-C6), degree of unsaturation, and oxidation of the carbon ring, flavonoids can be divided into various classes, such as chalcones, flavanones, flavonols, flavones, isoflavones, flavan-3-ols, and anthocyanidins (Figure 1) [3, 4].

These hydroxylated substances occur in all plants and are widely distributed in tissues and various plant parts, including roots, bark, leaves, flowers, fruits, and seeds. In plants, flavonoids play essential roles in several biological processes, such as protection against biotic and abiotic stresses, as well as defense against herbivores [5]. Due to the benefits they provide to plants, there has also been growing interest in their potential health benefits for humans. Therefore, the potential application of these natural products has been investigated in the pharmaceutical, medicinal, nutraceutical, and cosmetic fields [6].

Recently, a broad range of biological properties of flavonoids has been reported in the scientific literature, particularly antibacterial activity [7]. The emergence and increasing occurrence of multidrug resistance (MDR) and extensive drug resistance (XDR) among clinical isolates represent a serious public health threat in the 21st century [8, 9]. Various microorganisms have developed different resistance mechanisms to conventional antibiotics, driven by factors such as the excessive and improper use of these drugs in various contexts, including inappropriate prescriptions in hospital settings [10].

As a result, the reduced efficacy of these drugs has led to the increased use of last-resort agents, such as carbapenems. This practice has contributed to the emergence of multidrug-resistant pathogenic microorganisms, especially Gram-negative bacteria, such as *Klebsiella pneumoniae*, *Escherichia coli*, *Pseudomonas aeruginosa*, and *Acinetobacter baumannii*, that have acquired carbapenemase genes and are responsible for several severe infections in both hospital and community settings [11]. In addition to producing enzymes like beta-lactamases and carbapenemases, Gram-negative bacteria possess an outer membrane made of lipopolysaccharides (LPS) that serves as a protective barrier, hindering the entry of antibiotics and making the treatment of various diseases even more challenging [12, 13].

Consequently, this situation has rendered even the latest-generation medications obsolete, making therapeutic options scarce and complicating the treatment of infections caused by a wide range of Gram-negative bacteria. This results not only in increased morbidity and mortality associated with these diseases but also in significant economic costs due to the need for prolonged treatments and the use of last-line therapies, imposing a heavy economic burden on healthcare systems [14, 15].

Recent advancements point to a growing interest in developing effective strategies to control the resistance of these microorganisms, including the development of new antibacterial agents. Due to their ability to inhibit the growth of different microorganisms, flavonoids demonstrate significant efficacy in combating bacterial infections. These compounds can act through various mechanisms, such as altering membrane permeability, interfering with energy metabolism, inhibiting enzymes, and preventing biofilm formation [16, 17]. However, the inhibitory activities of plant-derived flavonoids against Gram-negative bacteria are still underreported.

Thus, the present study was aimed at reviewing and compiling updated information on the antibacterial activity of flavonoids isolated from different plant species against Gram-negative bacteria. To this end, this review sought not only to highlight the efficacy of flavonoids but also to identify gaps in the literature and propose directions for future advancements in understanding the antibacterial potential of flavonoids.

In this review, the methodological strategy employed was designed to ensure a comprehensive and up-to-date analysis of the antibacterial activity of plant-derived flavonoids against Gram-negative bacteria. The bibliographic research was conducted in the following databases: ScienceDirect, PubMed, SciELO, LILACS, and Web of Science. The search was performed using keywords such as “Gram-negative,” “flavonoids,” “isolated flavonoids,” “flavonoids as antibacterial agents,” and “antibacterial activity,” with these terms being searched individually or in combination.

The preliminary literature analysis resulted in the identification of 1958 publications, with the following distribution among the databases: 1223 from ScienceDirect, 394 from PubMed, 323 from Web of Science, 9 from SciELO, and 9 from LILACS. However, some articles were indexed in two or more databases. To ensure that the selected research was relevant and of high quality and to avoid duplication of studies, the data were subjected to a screening process based on inclusion and exclusion criteria. The inclusion criteria were as follows: only publications in English, investigations on the antibacterial activity of flavonoids isolated from plant species, and the action of flavonoids as antibacterial agents against Gram-negative strains. In addition, a publication time frame was defined, limiting articles to the last 22 years (2004–2025).

Thus, studies written in languages other than English, those outside the time frame (2004–2025), and those examining flavonoids only in combination with other compounds were excluded from the research. Furthermore, studies that did not use isolated flavonoids but merely flavonoid-enriched fractions were also excluded. Moreover, this review did not consider theses, dissertations, or publications with low reliability, such as drafts or preprints. After applying these criteria, 81 articles were selected for inclusion in the study, while the remaining 1876 publications that did not meet the defined criteria were excluded.

## 2. Antibacterial Activity of Flavonoids

Although the inhibitory activities of plant flavonoids against Gram-negative bacteria are less frequently reported than against Gram-positive bacteria, several studies have demonstrated their antibacterial efficacy against a range of pathogenic microorganisms, including multidrug-resistant bacteria (Table S1, available in the Supporting Information).

### 2.1. Antibacterial Activity of Chalcones

Chalcones derived from *Treculia obovoidea*, such as 4,2⁣′,4⁣′-trihydroxychalcone and its prenylated variant, showed promising activity against enteric bacteria, with effective inhibition of the growth of *Proteus vulgaris*, *Shigella flexneri*, *Salmonella typhi*, *Citrobacter freundii*, and *Enterobacter cloacae* (MICs ranging from 4.88 to 78.12 *μ*g/mL) [18]. In this study, the ability to interact with bacterial cell walls was suggested as a possible mechanism of action, although this hypothesis was not experimentally confirmed.

A study conducted by Mbaveng et al. [19] investigated four flavonoids isolated from the stems of *Dorstenia barteri*, revealing that the prenylated chalcones isobavachalcone (MIC 0.3–39.1 *μ*g/mL) and kanzonol C (MIC 4.9–39.1 *μ*g/mL) demonstrated the highest efficacy against all tested strains, including *S. typhi* and *K. pneumoniae*, while other compounds such as 4-hydroxylonchocarpin exhibited selective activity against certain bacteria. Although interaction with bacterial cell wall structures was also suggested as a mechanism of action, further mechanistic elucidation remains lacking.

In other studies, such as that by Inamullah et al. [20], the flavonoids colucins A and B, isolated from *Colutea armata*, exhibited significant antibacterial activity against *E. coli*, *P. aeruginosa*, *P. pseudomallei*, and *S. typhi*, while colucone displayed moderate activity. Additionally, Jamil et al. [21] investigated the potential of chalcones isolated from species of the genus *Artocarpus*, and the results revealed that isobavachalcone demonstrated notable activity against *E. coli* and *P. putida* (MIC of 0.45 mg/mL), whereas 2⁣′,4⁣′-dihydroxy-3,4-(2⁣^″^,2⁣^″^-dimethylchromeno)-3⁣′-prenyldihydrochalcone showed activity limited to *E. coli* (inhibition zone [IZ] of 7.25 mm). The prenylated chalcone flemichin D (MIC > 150* μ*g/mL) and the hydroxylated chalcone 2⁣′,4⁣′-dihydroxychalcone (MIC 62–250 *μ*g/mL) exhibited activity against *E. coli* and *P. aeruginosa* [22, 23].

Based on the studies reviewed, it is evident that chalcones exhibit antibacterial activity against Gram-negative bacteria; however, their efficacy varies according to structural modifications, especially due to the presence of hydroxyl and prenyl groups. Most studies suggest that the mechanism of action of compounds in this subclass may involve interaction with the bacterial cell wall, although further experimental studies are still needed to confirm this hypothesis (Table 1).

### 2.2. Antibacterial Activity of Flavanones

Based on the studies analyzed (Table 2), flavanones have been identified as promising compounds in the fight against Gram-negative bacteria, standing out due to the variability of their structures and potential mechanisms of action. Flavanones such as naringenin, pinocembrin, and 7-*O*-methyleriodictyol, isolated from species of the *Heliotropium* genus, showed significant activity against *E. cloacae*, *E. coli*, *K. pneumoniae*, and *P. mirabilis*, with MICs ranging from 0.25 to 4 *μ*g/mL [24]. The authors suggest that the efficacy of these compounds is associated with their physicochemical properties, such as lipophilicity and diffusion coefficient, as well as the presence of hydroxyl groups, which may have facilitated penetration into the bacterial membrane.

Research conducted by Mbaveng et al. [25] investigated nine prenylated flavonoids isolated from *Dorstenia mannii*, including dorsmanins A–G, *I*, and 6,8-diprenyleriodictyol, which showed strong inhibition of multidrug-resistant bacteria such as *Providencia stuartii*, *P. aeruginosa*, *K. pneumoniae*, *Enterobacter aerogenes*, and *E. coli*. Among them, dorsmanin C and dorsmanin F stood out, with MICs of 4 *μ*g/mL, surpassing even reference antibiotics.

The prenylated flavanone 8,3⁣′-diprenyl-5,7,4⁣′-trihydroxyflavanone, isolated from *Flemingia strobilifera*, also demonstrated potent activity against *P. aeruginosa* and *E. coli* (MIC of 17 *μ*g/mL) [26]. Similarly, 6-prenylpinocembrin, derived from *Pseudarthria hookeri*, exhibited MIC values ranging from 4 to 32 *μ*g/mL against the aforementioned bacteria and *K. pneumoniae* [27].

In the study by Thongnest et al. [22], it was observed that the prenylated flavanone lupinifolinol, isolated from the roots of *Eriosema chinense*, inhibited the growth of *E. coli*, *K. pneumoniae*, and *P. aeruginosa*, though with moderate activity (MIC > 150* μ*g/mL). The presence of prenyl groups was associated with this activity, but no in-depth investigation into the mechanisms of action was conducted. Flavanones isolated from *Artocarpus anisophyllus* and *A. lowii*, such as 5,7-dihydroxy-4⁣′-methoxy-6-prenylflavanone, also showed activity against *E. coli* and *P. putida*, with an MIC of 0.90 mg/mL and IZs of 9.0 and 9.25 mm, suggesting moderate activity [21]. Chatzopoulou et al. [28] reported that among the various flavonoids extracted from *Origanum dictamnus*, only eriodictyol was effective against *P. aeruginosa* (MIC of 0.174 mmol/mL), reinforcing the idea that certain flavanones may be particularly effective against specific strains of Gram-negative bacteria.

Most flavanones demonstrated good antibacterial activity, especially against *E. coli*, which was one of the main strains tested. Proposed mechanisms of action include disruption of membrane integrity, inhibition of nucleic acid synthesis, and alterations in energy metabolism. However, many of these hypotheses remain without direct experimental confirmation, indicating a critical gap in mechanistic elucidation. Therefore, integrated approaches such as molecular biology studies, electron microscopy, and molecular modeling are still needed to confirm the bacterial targets of these compounds. Furthermore, synergy tests with conventional antibiotics, toxicity studies, and pharmaceutical formulation are essential steps toward the development of flavanones as therapeutic agents.

### 2.3. Antibacterial Activity of Flavones

The antibacterial potential of flavones has also been highlighted in several studies (Table 3), particularly against multidrug-resistant Gram-negative bacteria. Compounds such as 6-methoxy-7-methyl-luteolin, hispidulin, hispidulin-7-*O-β*-D-glucuronopyranoside, apigenin-7-*O-β*-D-glucuronopyranoside, and apigenin-7-*O*-(6-methoxy)-*β*-D-glucuronopyranoside, isolated from *Centaurea pungens*, exhibited activity against *P. aeruginosa* (MIC: 100 *μ*g/mL), possibly due to membrane disruption [29].

Glycosylated flavonoids from *Graptophyllum grandulosum*, such as chrysoeriol-7-*O-β*-D-xyloside, and its apiofuranosyl and rhamnopyranosyl derivatives, showed activity against multidrug-resistant *Vibrio cholerae*, with some MICs comparable to vancomycin [30]. The loss of intracellular material, evidenced by the increase in optical density at 260 nm, suggests that the mechanism of action of these compounds may be related to their ability to destabilize bacterial membranes and induce cell lysis.

Similarly, flavonoids from *Mangifera indica* reduced growth in all tested Gram-negative strains, with 5-hydroxy-3-(4-hydroxyphenyl)pyrano[3,2-g]chromen-4(8H)-one being the most effective [31]. C-glycosyl flavones from *Rhynchosia beddomei*, such as 5,7,3⁣′,4⁣′-tetrahydroxy-6-C-*β*-D-glucopyranosyl flavone (IZ of 19.1 and 14.8 mm) and isovitexin (17.5 and 12.8 mm), inhibited *P. aeruginosa* and *E. coli*. The study suggests that the presence of glycosidic groups in flavonoids may contribute to their antibacterial activity, and molecular docking indicated strong binding to *P. aeruginosa* peptide deformylase (PaPDF), suggesting enzymatic inhibition [32].

Several flavonoids displayed particularly low MICs: chrysoeriol and luteolin (1–6.25 *μ*g/mL) against *E. coli*, *K. pneumoniae*, and *P. aeruginosa* [33, 35]; luteolin-4⁣′-neohesperidoside from *Phyllanthus emblica* (MICs: 26.66 and 53.33 *μ*g/mL; IZ: 20.6 and 18.0 mm for *E. coli* and *K. pneumoniae*, respectively), showing synergism with gentamicin [17]. 5,7,4⁣′-Trimethoxyflavone from *Praxelis clematidea* was active against multidrug-resistant *E. coli* and *P. aeruginosa* (MIC: 128 *μ*g/mL) [36], while 3⁣′,4⁣′,7-trihydroxyflavone from *Rhus verniciflua* inhibited *E. coli* (MIC: 32 *μ*g/mL) [37]. Licoflavone C (*Retama raetam*) and 5-hydroxy-3,7-dimethoxyflavone-4⁣′-*O-β*-glucopyranoside (*Calotropis procera*) also showed relevant activity, with IZs of 22 and 20 mm and MICs ranging from 0.08 to 0.64 mg/mL [39].

Compounds from *Adenium obesum*, such as 5,7,3⁣′,4⁣′-tetrahydroxyflavone and its hexahydroxylated analogue (e.g., 3,5,7,3⁣′,4⁣′,5⁣′-hexahydroxyflavone) from *Adenium obesum*, showed IZs up to 14 mm against *P. vulgaris* [40]. Vignafuran from *Spatholobus parviflorus* inhibited *P. aeruginosa*, *E. coli*, and *S. typhimurium* (MICs: 32–128 *μ*g/mL) [41]. The flavone 3,4⁣′,5-trihydroxy-3⁣′,7-dimethoxyflavone from *Dodonaea angustifolia* showed potent activity against *E. coli* (MIC < 31.25* μ*g/mL) [42], while among the flavonoids isolated from *Orthosiphon aristatus*, only oraristatinoside A was active (MIC: 150 *μ*M/mL) [43]. Flavones from *Broussonetia papyrifera* inhibited oral and intestinal pathogens, with MICs from 3.9 to 250 ppm [44]. Variable activity against different Gram-negative strains was also observed for genkwanin and 5-hydroxy-7,4-dimethoxyflavone (MICs of 25–100 *μ*g/mL) from *Combretum erythrophyllum* [16], as well as for luteolin and diosmetin (MICs 1–2.85 mg/mL) from *Penstemon campanulatus* [34].

Pseudarflavone A from *P. hookeri* exhibited MICs between 4 and 32 *μ*g/mL against *E. coli*, *P. aeruginosa*, and *K. pneumoniae* [27]. The bacteriostatic effect observed suggests a mechanism that inhibits bacterial growth without causing immediate cell death. 3⁣′,4⁣′,7-Trihydroxyflavone from *Myristica fragrans* also demonstrated broad activity (MICs: 4–128 *μ*g/mL) and synergism with antibiotics and efflux pump inhibitors (FIC: 0.5–< 0.062) such as PA*β*N (phenylalanine-arginine-*β*-naphthylamide) [45].

Clerodendronone 1a, clerodendronone 1b, and 5,7-dihydroxy-4⁣′-methoxyflavone from *Clerodendrum formicarum* inhibited *S. flexneri* (MIC: 62.5 *μ*g/mL) [46]. Flavonoids from *Pistacia integerrima* (e.g., 3,5,7,4⁣′-tetrahydroxyflavanone, naringenin, 3,5,4⁣′-trihydroxy-7-methoxyflavanone, and sakuranetin) presented IZs of 12–24 mm and MICs ranging from 34 to 104 mg/mL against *E. coli* [47]. Vernoguinoflavone from *Vernonia guineensis* exhibited moderate to low activity against *E. coli*, *S. Muenchen*, and *S. typhimurium* [35].

Gliricidin 7-*O*-hexoside from *Asplenium nidus* showed significant activity against *P. mirabilis*, *P. vulgaris*, and *P. aeruginosa*, with MIC_50_ values between 0.005 and 6.0 *μ*g/mL, comparable to amoxicillin [48]. In contrast, norwogonin from *Scutellaria baicalensis* had limited activity against *A. baumannii* (MIC: 128 mg/mL and MBC: 256 mg/mL), without synergism [49], suggesting that the synergistic effect between flavonoids and antibiotics may be limited depending on the nature of the flavonoid and the bacterial strain.

Other promising flavones include isocytisoside from *Aquilegia vulgaris* (MICs: 125–250 *μ*g/mL) [50]; flavonoids from *Iris tenuifolia*, such as 5-methoxy-6,7-methylenedioxy-4-*O*-2⁣′-cycloflavan, 5,7,2⁣′,3⁣′-tetrahydroxyflavanona, and 5,2⁣′,3⁣′-trihydroxy-6,7-methylenedioxyflavanona, which showed activity against *Mycobacterium vaccae* (IZ of 16–23 mm) [51]; and 5-carboxymethyl-4⁣′,7-dihydroxyflavanone derivatives, active against *E. coli* and *Helicobacter pylori* (MICs: 12.5–50 *μ*g/mL; MBCs: 25–100 *μ*g/mL) [52].

Luteolin 7-*O-β*-D-glucoside from *Cephalaria elmaliensis* inhibited several Gram-negative strains (MIC: 16 *μ*g/mL) [53]. Flavones from *Artocarpus* spp., including artocarpin, also demonstrated activity (MIC/MBC: 0.45–0.90 mg/mL) [21]. Some compounds showed limited efficacy, such as 5-hydroxy-6,7,8,2⁣′,4⁣′-pentamethoxyflavone from *Artemisia kulbadica* (MIC: 256–512 *μ*g/mL) [54] and apigenin and chrysoeriol from *Chresta scapigera*, which required high concentrations for activity (2500 *μ*g/mL) [55], highlighting the need for structural optimization.

In summary, flavones demonstrate considerable antibacterial potential, particularly those with hydroxyl, methoxyl, and glycosyl substitutions. Their mechanisms may involve membrane destabilization, enzymatic inhibition, and synergism with antibiotics. However, further molecular-level studies are needed to elucidate these mechanisms and optimize bioactivity.

### 2.4. Antibacterial Activity of Flavonols

Flavonols such as 3-*O*-methylquercetin and 3,3⁣′-di-*O*-methylquercetin from *Inula viscosa* inhibited *S. typhimurium* with an MIC of 125 *μ*g/mL. This effect is likely due to their ability to disrupt bacterial cell integrity and viability by altering membrane permeability [56]. This mechanism was also suggested to explain the antibacterial activity of morin, isolated from *Elaeagnus umbellata* (MIC: 21–44 *μ*g/mL) [57]. Other flavonols demonstrating strong antibacterial potential include 3-*O*-methylgalangin, isolated from *Heliotropium* species, with MICs between 0.25 and 1 *μ*g/mL against *E. coli*, *K. pneumoniae*, *P. mirabilis*, and *E. cloacae* [24].

Additionally, kaempferol-3-*O-α*-L-rhamnopyranoside and quercetin-3-*O-α*-L-rhamnopyranoside from *Albizia chinensis* produced IZs of 16–17.5 mm against *E. coli* [58]. Flavonols from *Diplotaxis* species, such as isorhamnetin-3-*O*-*α*-L-glucopyranoside and rhamnetin-3,3⁣′-di-*O-β*-D-glucopyranoside, also exhibited notable antibacterial activity (IZ: 12.5–17.6 mm). According to the authors, the structure–activity relationship may be associated with the antibacterial potential of these flavonols [59]. Further examples include kaempferol from *Vismia laurentii*, which demonstrated a broad spectrum of activity (MIC: 4.88–78.12 *μ*g/mL; MBC: 9.76–78.12 *μ*g/mL) [60].

Several glycosylated quercetins showed promising activity: quercetin-7-O-methylether (IZ: 16.8–20.4 mm), quercetin-3⁣′-O-*β*-D-glucopyranoside (21–26 mm) [32, 61], and quercetin-3-O-*α*-L-rhamnopyranoside-2⁣^″^-gallate from *Salvia leucantha*, active against *E. coli* and *P. aeruginosa* (IZ: 2–19 mm) [62]. Flavonols from *Argyreia speciosa*, with acylated or sulfated groups, showed potent activity against *K. pneumoniae* (MIC: 2 *μ*g/mL) and *E. coli* (MIC: 62.5 *μ*g/mL), with a 70% survival rate in infected mice [63].

Compounds from *Entada abyssinica* (quercitrin and quercetin-3-*O-β*-D-glucosyl (1 → 4)-*α*-l-rhamnoside) showed MICs of 3.12–50 *μ*g/mL, with quercitrin being particularly effective against *S. typhimurium* [64]. Contrastingly, quercitrin from *Gynotroches axillaris* exhibited only moderate activity (MICs and MBCs: 450–900 *μ*g/mL) [65]. Similarly, flavonols from *Alternanthera maritima*, such as quercetin 3-methyl ether, kaempferol, and isorhamnetin-3-*O*-robinobioside, and quercetin-3-*O*-rutinoside had higher MICs (100–500 mg/mL) [66], indicating variability among structurally related compounds.

From *Combretum erythrophyllum*, rhamnocitrin, quercetin-5,3-dimethylether, and rhamnazin exhibited MICs of 25–100 *μ*g/mL and low cytotoxicity [16]. Isorhamnetin 3-*O-β*-D-glucoside and isorhamnetin 3-O-*β*-D-rutinoside inhibited *E. coli* (MICs: 10–26 *μ*M) [67]. Kaempferol 3-*O*-[3-*O*-acetyl-6-*O*-(E)-p-coumaroyl]-*β*-D-glucopyranoside and astragalin (kaempferol 3-*O-β*-D-glucoside), isolated from *Scabiosa hymettia*, showed potential against *P. aeruginosa*, *E. coli*, *E. cloacae*, and *K. pneumoniae* (IZ: 10–13 mm) [68]. These findings underscore the significance of flavonoid structure, with specific modifications enhancing antibacterial activity.

Furthermore, flavonoids such as kaempferol-3-*O*-rutinoside, isorhamnetin-3-*O*-rutinoside, and quercetin-3-*O*-rutinoside from *C. procera* exhibited MICs of 0.08–0.64 mg/mL and IZs of 8.5–20.5 mm [39]. The authors suggest that the activity of these compounds may be related to their ability to form complexes with extracellular and soluble proteins, such as bacterial glutamate and phosphate, leading to peptidoglycan disruption and altered membrane permeability. Additionally, the compounds may inhibit vital enzymes like cytochrome P450-dependent oxidases. The presence of hydroxyl groups could also influence their antibacterial efficacy. However, further investigations are needed to confirm these theories.

Studies like that of Tebou et al. [69] highlight that the presence of hydroxyl groups and sugars directly influences the bioactivity of flavonols, with these compounds potentially interacting with bacterial proteins and cell walls. In this study, quercetin-3-*O-β*-D-glucopyranoside was effective (MIC of 32–64 *μ*g/mL), eliminating *V. cholerae* and *S. flexneri* after 6 h. Quercetin-3-O-glucoside (*Acacia polyacantha*) and quercetin-7-O-rutinoside (*Asplenium nidus*) also displayed notable activity [48, 70].

Moderate antibacterial effects were noted for isorhamnetin 3-*O-β*-rutinoside and quercetin 3-*O-β*-rutinoside from *Galium brunneum* (IZ: 7–10.3 mm) [71], quercetin-3-*O-β*-D-galactopyranoside from *Calendula stellata* (MICs: 125–250 *μ*g/mL) [72], and quercetin from *Buddleja salviifolia* (MIC: ~125 *μ*g/mL) [73]. Macaragin and quercetin from *Macaranga conglomerata* showed highly variable MICs (1–500 mg/mL), suggesting structure-dependent efficacy [74]. Furthermore, the antibacterial potential of quercetin has been confirmed by other studies against *Salmonella Muenchen* [35] and *E. cloacae* [34]. In addition, research by Elumalai et al. [75] validated the activity of this compound (MIC of 125 *μ*g/mL), although the addition of metals such as zinc did not necessarily enhance its effect, suggesting that metal–flavonoid complexes warrant further investigation.

Myricetin-3-*O*-(3⁣^″^-*O*-methyl)-*α*-L-rhamnopyranoside from *Manilkara hexandra* demonstrated strong inhibition (MICs: 0.98–3.9 *μ*g/mL; IZ: 20.3–22.9 mm), comparable to gentamicin [76]. Pollenitin-3-I-D-mannopyranoside (ephedroside B) from *Ephedra sinica* showed potent activity against *P. aeruginosa* (MIC of 0.105 mM) [77]. Flavonoids from *Ruta chalepensis* (rutin, rutin 3-methyl ether, and 6-hydroxy-rutin 3,7-dimethyl ether) had MICs of 0.25–0.5 mg/mL [78], while tiliroside from *C. elmaliensis* exhibited MIC of 16 *μ*g/mL [53].

Despite these findings, not all flavonols showed significant activity. Flavonols from *Lannea alata* exhibited limited efficacy against Gram-negative bacteria, with only lanneaflavonol showing relevant results, comparable to tetracycline against *P. aeruginosa* [79]. Similarly, fisetin and fustin from *R. verniciflua* were effective against *E. coli* (MICs of 8 and 63 *μ*g/mL, respectively) but inactive against *Salmonella typhimurium* [37], suggesting a selective mode of action. In clinical strain studies, ericoside displayed enhanced activity against multidrug-resistant *E. coli* (MIC: 64 *μ*g/mL), despite moderate effects on standard strains (MIC: 128 *μ*g/mL). The authors proposed that antibacterial activity may be linked to interactions with bacterial membranes, although a more thorough elucidation of these mechanisms is clearly needed.

In summary, flavonols exhibit a broad antibacterial spectrum, with MICs ranging from low microgram/milliliter to milligram/milliliter levels, especially against *E. coli* and *P. aeruginosa*. Structural elements such as hydroxyl groups, glycosides, methylations, and sulfations play a key role in modulating activity. Proposed mechanisms include disruption of membrane integrity, inhibition of essential enzymes, and interaction with bacterial proteins. Nevertheless, further studies addressing structure–activity relationships, in vivo validation, toxicity, and formulation are essential to advance flavonols as viable antibacterial agents (Table 4).

### 2.5. Antibacterial Activity of Isoflavonoids

Although exhibiting significant variability in potency, some isoflavonoids have shown promising antibacterial activity against Gram-negative bacteria (Table 5). In *Hypericum oblongifolium*, the isoflavone 7,4⁣′-dihydroxy-5,3⁣′-dimethoxyisoflavone exhibited significant IZs against *S. typhi*, *E. coli*, and *P. aeruginosa* (17–23 mm), and molecular docking studies suggest its action may be related to the inhibition of key enzymes such as lipoxygenase (5-LOX) and GlcN-6-P synthase [61]. Genistin, a prenylated isoflavone extracted from *Flemingia strobilifera*, also showed potential against *P. aeruginosa* (MIC 136 *μ*g/mL) and *E. coli* (MIC 146 *μ*g/mL) [26], while compounds derived from *Uraria picta* demonstrated greater efficacy (MIC 12.5–100 *μ*g/mL) against *E. coli* and *P. vulgaris* [81].

Studies conducted by Kuete et al. [19] revealed that isoflavonoids such as alpinumisoflavone, genistein, and laburnetin, isolated from *Ficus chlamydocarpa*, exhibited selective activity against *E. cloacae*, *M. morganii*, and *Proteus mirabilis*. The proposed mechanism of action involves the complexation of these compounds with the bacterial cell wall, leading to reduced microbial viability. A similar mechanism was suggested for gancaonin Q, stipulin, angusticornin B, and bartericin A, isolated from *Dorstenia angusticornis*, which showed strong activity (MIC 0.31–78.12 *μ*g/mL), with angusticornin B and bartericin A being as effective as reference antibiotics [82].

Prenylated isoflavones extracted from *Erythrina caffra*, such as abyssinone V 4⁣′-*O*-methyl ether, 6,8-diprenylgenistein, alpinumisoflavone, and burtinone, showed good results against *E. coli* and *K. pneumoniae* (MIC 3.9–62 *μ*g/mL), suggesting that prenylation may increase lipophilicity and facilitate membrane penetration [83]. Similarly, isoflavones from *Millettia extensa* and *Spatholobus parviflorus* demonstrated moderate activity against *P. aeruginosa*, *S. typhimurium*, and *Serratia marcescens*, with MICs ranging from 64 to 128 *μ*g/mL [41, 84].

The flavonoid derrone, isolated from the flowers of *Retama raetam*, stood out due to its significant activity against *E. coli* and *P. aeruginosa*, with MICs ranging from 7.81 to 15.62 *μ*g/mL and IZ of 19 and 16 mm, respectively. The study suggested that its mechanism of action might involve the formation of protein complexes and interaction with the bacterial cell wall [38]. In contrast, compounds from *Crotalaria lachnophora*, such as lachnoisoflavone A and B, exhibited moderate IZs (7.0–8.3 mm) against *E. coli* and *K. pneumoniae* [85], and isoflavonoids from *Erythrina lysistemon*, such as wighteone and 4⁣′,5,7-trihydroxy-6-(2⁣^″^-hydroxy-3⁣^″^-methylbut-3⁣^″^-enyl)isoflavone, displayed only limited activity against *E. coli* [86].

Although many of these compounds demonstrate potential, particularly those with prenyl or glycosidic modifications, variations in assay methodologies, tested concentrations, and bacterial strains make direct comparisons across studies challenging. Furthermore, few works thoroughly explore molecular mechanisms of action, limiting the understanding of how these flavonoids interact with specific bacterial targets.

Thus, while isoflavonoids exhibit antibacterial activity, further investigation is needed for their progression toward clinical application. Such studies should focus on pharmacokinetic properties, toxicity, stability, and, most importantly, their molecular mechanisms of action. In vivo studies and clinical trials are also essential to validate their use as alternative antimicrobial agents.

### 2.6. Antibacterial Activity of Flavanols

Another subclass that has demonstrated antibacterial activity against Gram-negative bacteria is that of flavanols (Table 6), although they have shown varying degrees of efficacy. The flavanol piafzelechin, isolated from *Ficus cordata*, exhibited inhibitory activity against a wide range of microorganisms, including *E. coli*, *S. dysenteriae*, *P. mirabilis*, *K. pneumoniae*, *P. aeruginosa*, and *S. typhi*, with MICs ranging from 19.53 to 78.12 *μ*g/mL [19]. This broad spectrum suggests that piafzelechin may act through nonspecific mechanisms, such as destabilization of the bacterial membrane or interference with essential proteins.

In another study, Kanwal et al. [31] reported that flavonoids isolated from *Mangifera indica*, particularly (–)-epicatechin (2-(3,4-dihydroxyphenyl)-3,4-dihydro-2H-chromen-3,5,7-triol), effectively inhibited the growth of tested Gram-negative strains. The proposed mechanisms include inhibition of nucleic acid synthesis, disruption of the cytoplasmic membrane, and interference with energy metabolism, common pathways through which flavonoids exert antibacterial effects. However, these hypotheses were not confirmed by biochemical or molecular assays, indicating a significant methodological gap.

Flavonoids such as epicatechin and catechin, extracted from *Schotia latifolia*, were also effective against *E. coli*, *K. pneumoniae*, and *P. aeruginosa*, with MICs ranging from 62.5 to 125 *μ*g/mL [87]. Additional studies, such as that by Pretorius et al. [88], reinforce this potential by reporting that epicatechin isolated from *Euclea crispa* showed a 12 mm IZ against *K. pneumoniae*. Although further studies are needed, these findings suggest that catechin-type structures possess consistent antimicrobial properties.

Research conducted by Guyasa et al. [90] with compounds from *Embelia schimperi* showed that both epicatechin and a flavan derivative moderately inhibited *K. pneumoniae*, *E. coli*, and *S. dysenteriae*, with IZ ranging from 6 to 12 mm for epicatechin and 10–13 mm for the flavan derivative. Although these values are not high, the consistent observation of activity against resistant strains suggests pharmacological relevance. Similarly, Ahmed et al. [65] confirmed the antibacterial activity of epicatechin isolated from *Gynotroches axillaris* against *E. coli* and *P. aeruginosa*.

A more recent study by Tonga et al. [89], using compounds isolated from *Staudtia kamerunensis*, demonstrated that epicatechin exhibited MICs between 15.625 and 62.5 *μ*g/mL for most tested bacteria, although it was less effective against *E. coli* and *K. pneumoniae* (MIC of 250 *μ*g/mL). Additionally, studies involving epicatechin extracted from *Maytenus buchananii* and *Acacia polyacantha* reported activity against *V. cholerae*, *S. flexneri*, *E. aerogenes*, *K. pneumoniae*, and *P. aeruginosa*, with MICs ranging from 32 to 256 *μ*g/mL [69, 70].

Epigallocatechin-3-gallate (EGCG), a widely studied derivative isolated from the bark of *Erythrina livingstoniana*, demonstrated activity against *Stenotrophomonas maltophilia*, though with high MICs (256 mg/L in agar dilution and 512 mg/L in broth microdilution) and an MBC of 512 mg/L, indicating moderate activity [91] Although EGCG is recognized for multiple bioactivities, in this study, its antimicrobial performance against Gram-negative bacteria was limited, which may be related to factors such as poor cell wall penetration or rapid metabolic degradation.

In summary, flavanols exhibit antibacterial potential, particularly epicatechin and its derivatives. However, direct comparison between these compounds is challenging due to variability in results across studies, which may be attributed to structural differences among flavanols as well as methodological inconsistencies. It is commonly suggested that flavanols act by altering bacterial membrane permeability, forming complexes with enzymes, or interfering with critical metabolic pathways. Nevertheless, despite advances, the literature still lacks studies that clearly elucidate their mechanisms of action. Therefore, to harness the therapeutic potential of these compounds, further investigations are required regarding their molecular targets, in vivo stability, bioavailability, and synergy with conventional antibiotics, as well as pharmacodynamic and toxicological studies.

### 2.7. Antibacterial Activity of Flavanonols and Tetraflavonoids

The ethylated flavanonol extracted from *M. hexandra*, 3-*O*-ethyl-dihydroquercetin, also demonstrated potent activity against *P. aeruginosa*, *E. coli*, *S. typhimurium*, and *K. pneumoniae*, with IZ ranging from 20.3 to 22.9 mm and MICs varying from 0.98 to 3.9 *μ*g/mL [76]. These results indicate that structural modifications such as ethylation can enhance the bioactivity of flavanonols, bringing them closer to the efficacy of conventional antibiotics.

Although more moderate compared to the previously mentioned compound, the inhibition halos also suggest relevant antibacterial activity for a dihydroflavonol isolated from *Tamarix nilotica*, which showed inhibition against *E. coli* and *S. typhi*, with IZs of 16 and 14 mm, respectively [92]. On the other hand, the glycosylated flavanonol taxifolin 3-*O-α*-L-rhamnopyranoside, isolated from *E. mannii*, showed only moderate activity against *E. coli*, *E. aerogenes*, *K. pneumoniae*, and *P. stuartii*, with a MIC of 128 *μ*g/mL [80].

Regarding tetraflavonoids, Bitchagno et al. [93] investigated the compounds lemairone A and lemairone B, isolated from the leaves of *Zanthoxylum lemairei*. Both exhibited moderate to low activity, with *E. coli* being the most sensitive strain to the compounds. The MBC revealed that only lemairone B showed a moderate bactericidal effect (MBC 256 *μ*g/mL), which may not be sufficiently potent to be considered bactericidal at lower concentrations. However, this difference between the compounds suggests that small structural variations among tetraflavonoids can significantly impact their activity.

In summary, few studies have addressed the antibacterial potential of these subclasses (Table 7). Among the compounds studied, 3-*O*-ethyl-dihydroquercetin was the most therapeutically promising against Gram-negative bacteria. Nevertheless, similar to the previously mentioned subclasses, significant gaps remain in understanding their mechanisms of action, as well as a lack of data regarding toxicity, stability, and synergy with conventional antibiotics, which limits their advancement toward clinical applications.

## 3. Mechanisms of Actions of Flavonoids

Secondary metabolites from plants, such as flavonoids, often exhibit variable activity against different types of bacteria. Furthermore, the mechanisms of action of these compounds can be complex and multifactorial in Gram-negative bacteria [94]. The diversification of mechanisms can occur through changes in the integrity of the cell wall and membrane, interference with efflux pumps, inhibition of energy metabolism, cell proliferation, nucleic acid synthesis, and protein synthesis. Additionally, they can reduce biofilm formation and cellular adhesion, attenuate pathogenicity, induce oxidative stress, and inhibit intracellular enzymes and the cell envelope, among others [87].

The presence of an outer cell membrane rich in LPS makes the discovery of effective antibacterial agents against Gram-negative bacteria a challenge [94]. However, some studies suggest that the inhibition of bacterial growth caused by flavonoids may occur due to interactions with the cell membrane. The bacterial cell membrane plays a crucial role in protecting the cell, functioning as a selective barrier that regulates the entry and exit of substances, as well as ensuring the structural and functional integrity of the cell [95]. In Gram-negative bacteria, in addition to the cytoplasmic inner membrane and the peptidoglycan layer, the presence of an outer membrane serves as an additional barrier, making it more difficult for small molecules to enter and, consequently, rendering these bacteria naturally more resistant to many antibiotics [24].

However, membrane alterations caused by antibacterial agents can lead to metabolic dysfunctions, compromising cellular homeostasis and causing the membrane to rupture, releasing cytoplasmic contents, which lead to cell death or bacterial inhibition [96].

This interaction with the cell membrane is considered one of the main mechanisms of action by which various antibacterial agents act to destroy or inhibit bacterial growth. However, systematic studies exploring the mechanism responsible for flavonoid–membrane interaction at the molecular level are still scarce, and this mechanism is not yet fully understood, leaving gaps in the current knowledge [97].

Studies have also suggested a relationship between the structure of flavonoids and their antibacterial activity. According to [98], the antibacterial activity of flavonoids from different classes may be linked to their structural properties, which enable them to interact effectively with biological targets, such as bacterial cell membranes, enzymes, and resistance mechanisms, such as efflux pumps. Therefore, the specificity of this interaction may vary based on the flavonoid structure and the composition of the bacterial cell wall. However, more detailed studies are still needed to understand how the chemical structure of flavonoids influences their interaction with these complex structures.

Another well-known mechanism by which bacteria reduce the effectiveness of antibacterial compounds is through the expression of efflux pumps, which actively expel antibiotics from the cell [99]. These pumps are proteins that play key roles in bacterial cells, such as protection against oxidative stress and biofilm stabilization. Some flavonoids have the ability to block or reduce the efficiency of these efflux pumps, as well as act synergistically with drugs, allowing antibiotics to accumulate inside the cell and restore their antibacterial activity [100].

Biofilm formation is also an important target of study in antibacterial activity. Bacterial biofilms grow as communities surrounded by a thick layer of self-produced exopolysaccharide (EPS) substances [101]. The presence of biofilms plays crucial roles in cell adhesion to surfaces and protection against adverse environmental factors, such as dehydration, immune attacks, and the action of antimicrobials. Thus, biofilms formed by pathogenic bacteria are responsible for causing various health issues, such as cystic fibrosis, kidney infections, prostatitis, and periodontitis [102, 103].

Although the literature reports a limited number of antibiofilm compounds, studies indicate that flavonoids are capable of reducing the gene expression of virulence factors associated with biofilm formation, acting on multiple targets and presenting different mechanisms, such as modulation of the quorum sensing system, reduction of bacterial adhesion and motility, blocking EPS production, and destabilizing biofilm structure [101, 104].

## 4. Open Questions and Further Studies

From the analyzed data, it is possible to verify that there are still gaps in current research regarding the antibacterial activity of flavonoids against Gram-negative bacteria. Although the growing literature on plant flavonoids has shown the promising potential of these compounds against certain pathogenic bacteria, most of the studies reviewed focused exclusively on the simple evaluation of the presence or absence of antimicrobial activity, without deeper investigation into the mechanisms of action, the clinical efficacy of flavonoids, or their toxicity.

It was observed that many of the studies only measured IZs or determined the MIC for different bacterial strains without exploring aspects such as the interaction between flavonoids and other substances, such as traditional antibiotics, bactericidal versus bacteriostatic effects, and the possibility of bacterial resistance. These initial studies are important for indicating antibacterial activity, but it is essential to understand the mechanisms of action of these compounds. There is still a need for more detailed approaches that investigate the pharmacokinetics, toxicity, and therapeutic potential of these flavonoids, especially in more complex animal models and clinical trials.

Considering that most experiments were performed in vitro, it is crucial to emphasize the importance of conducting in vivo investigations to validate their efficacy under real biological conditions and provide a broader view of their effects and safety. There is also a need to combine these experiments with molecular docking studies to provide a more comprehensive understanding of their antibacterial effects.

It should also be noted that a large part of the studies analyzed evaluated the antibacterial effects of flavonoid-rich extracts and fractions along with the flavonoids themselves, with relatively few studies focusing specifically on pure flavonoids, especially regarding activity against Gram-negative bacteria, given that most studies tested both classes of bacteria. This highlights a significant gap in the literature, as little is known about the isolated impact of pure flavonoids, specifically against Gram-negative bacteria, which limits the detailed understanding of the specific mechanisms of action of these compounds against these bacteria. Furthermore, most studies focus on a few species, such as *E. coli*, *P. aeruginosa*, and *K. pneumoniae*, making it interesting to explore the activity of flavonoids against a broader range of Gram-negative pathogens. The importance of conducting studies with flavonoids from different subclasses is also highlighted, considering the disparity in the number of compounds investigated among certain subclasses, such as flavones and flavanonols.

Considering that some flavonoids can enhance bacterial sensitivity and even reverse resistance to antibacterial agents, it is also important to conduct studies evaluating the antibacterial activity of flavonoids in combination with other substances. Examples of such substances include antibiotics that act through different mechanisms against Gram-negative bacteria, aiming to promote synergy and enhance the action of drugs in antibacterial therapy, as well as ensuring a reduction in the dose of both components. It is also important to mention the relevance of developing advanced formulations, such as the production of flavonoid nanoparticles, which can be used as a strategy to improve the delivery of these compounds to the site of infection, considering improved bioavailability and stability.

Expanding these investigations, as well as understanding how these compounds interact with bacterial membranes, inhibit essential enzymes, interfere with the biosynthesis of critical cellular components, and other mechanisms, could provide new information about the effectiveness of flavonoids. These actions contribute to the potential application as adjuvants or in the development of new, more effective, and targeted antibacterial agents that may help address the growing challenge of bacterial resistance.

## 5. Conclusions

In summary, through the studies analyzed, it was possible to observe that many flavonoids isolated from various plant species show promising roles in the fight against Gram-negative bacteria. However, despite advancements, it is important to highlight the gaps in current research regarding the understanding of their efficacy and potential use in developing new therapies effective against bacterial resistance.

A deeper understanding of their properties and the mechanisms underlying their activity, as well as interactions with other molecules, remains essential for the development of new antibacterial therapies. Furthermore, future investigations focusing on in vivo trials to validate the efficacy of compounds under real biological conditions, as well as the combination of flavonoids with conventional antibiotics to achieve synergy against resistant bacteria, are crucial for understanding the potential of flavonoids for effective clinical use. Additionally, there is a need for further studies focusing on the evaluation of pure flavonoids against Gram-negative bacteria, as well as the exploration of new flavonoids from plant sources against other Gram-negative bacteria.

## Figures and Tables

**Table 1:** Antibacterial activity of chalcones.

**Flavonoid name**	**Isolated from**	**Antibacterial effect against**	**References**
4,2⁣′,4⁣′-Trihydroxy-3-prenylchalcone	*T. obovoidea*	*P. vulgaris*, *S. flexneri*, *S. typhi*, *C. freundii*, *E. cloacae*	[18]
4,2⁣′,4⁣′-Trihydroxychalcone			
Isobavachalcone	*D. barteri*	*S. typhi*, *Shigella dysenteriae*, *S. flexneri*, *E. coli*, *E. aerogenes*, *E. cloacae*, *P. mirabilis*, *P. vulgaris*, *P. aeruginosa*, *K. pneumoniae*, *Morganella morganii*, *C. freundii*	[19]
Kanzonol C			
-4-Hydroxylonchocarpin		*S. flexneri*, *P. aeruginosa*, *P. vulgaris*, *P. mirabilis*, *E. cloacae*, *E. aerogenes*	
Colucone	*C. armata*	*E. coli*, *P. aeruginosa*, *P. pseudomallei*, *S. typhi*	[20]
Colucin A			
Colucin B			
Isobavachalcone	*A. anisophyllus*, *A. lowii*	*E. coli*, *P. putida*	[21]
2⁣′,4⁣′-Dihydroxy-3,4-(2⁣^″^,2⁣^″^-dimethylchromeno)-3-prenyldihydrochalcone		*E. coli*	
Flemichin D	*Eriosema chinense*	*E. coli*, *K. pneumoniae*, *P. aeruginosa*	[22]
2⁣′,4⁣′-Dihydroxychalcone	*Flourensia oolepis*	*E. coli*, *P. aeruginosa*	[23]

**Table 2:** Antibacterial activity of flavanones.

**Flavonoid name**	**Isolated from**	**Antibacterial effect against**	**References**
Naringenin	*H. filifolium*, *H. huascoense*, *H. sinuatum*	*E. cloacae*, *E. coli*, *K. pneumoniae*, *P. mirabilis*	[24]
Pinocembrin			
7-*O*-Methyleriodictyol			
Dorsmanin A, B, C, D, E, F, G, I	*D. mannii*	*P. stuartii*, *P. aeruginosa*, *K. pneumoniae*, *E. aerogenes*, *E. coli*	[25]
8,3⁣′-Diprenyl-5,7,4⁣′-trihydroxyflavanone	*F. strobilifera*	*P. aeruginosa*, *E. coli*	[26]
6-Prenylpinocembrin	*P. hookeri*	*E. coli*, *P. aeruginosa*, *K. pneumoniae*	[27]
Khonklonginol A	*E. chinense*	*E. coli*, *K. pneumoniae*, *P. aeruginosa*	[22]
Lupinifolinol			
Lupinifolin			
20-Hydroxylupinifolinol			
3,5,20,40-Tetrahydroxy-600,600-dimethylpyrano(200,300:7,6)-8-(3000,3000-dimethylallyl)flavone			
5,7-Dihydroxy-4⁣′-methoxy-6-prenylflavanone	*A. anisophyllus*, *A. lowii*	*E. coli*, *P. putida*	[21]
Eriodictyol	*O. dictamnus*	*P. aeruginosa*	[28]

**Table 3:** Antibacterial activity of flavones.

**Flavonoid name**	**Isolated from**	**Antibacterial effect against**	**References**
6-Methoxy,7-methyl-luteolin	*C. pungens*	*P. aeruginosa*	[29]
Hispidulin			
Hispidulin-7-*O*-*β*-D-glucuronopyranoside			
Apigenin-7-*O-β*-D-glucuronopyranoside			
Apigenin-7-*O*-(6-methoxy)-*β*-D-glucuronopyranoside			
Chrysoeriol-7-*O-β*-D-xyloside Luteolin-7-*O-β*-D-apiofuranosyl-(1 → 2)-*β*-D-xylopyranoside	*G. grandulosum*	*V. cholerae*	[30]
Chrysoeriol-7-*O-β*-D-apiofuranosyl-(1 → 2)-*β-D*-xylopyranoside			
Chrysoeriol-7-*O-α*-L-rhamnopyranosyl-(1 → 6)-*β*-D-(4⁣^″^-hydrogeno sulfate) glucopyranoside			
Pyranoflavone 5-hydroxy-3-(4-hydroxyphenyl)pyrano[3,2-g]chromen-4(8H)-one	*M. indica*	*E. coli*, *Azospirillum lipoferum*	[31]
5,7,3⁣′,4⁣′-Tetrahydroxy-6-C-*β*-D-glucopyranosyl flavone	*R. beddomei*	*P. aeruginosa*, *E. coli*	[32]
Isovitexin			
Chrysoeriol	*C. frutescens*	*E. coli*, *K. pneumoniae*, *P. aeruginosa*	[33]
Luteolin	*P. campanulatus*, *F. chlamydocarpa*	*E. coli*, *S. dysenteriae*, *P. mirabilis*, *K. pneumoniae*, *P. aeruginosa*, *S. typhi*, *M. morganii*, *C. freundii*, *E. cloacae*	[19, 34]
	*V. guineensis*	*E. coli*, *S. Muenchen*, *S. typhimurium*	[35]
Luteolin-4⁣′-neohesperidoside	*P. emblica*	*E. coli*, *K. pneumoniae*	[17]
5,7,4⁣′-Trimethoxyflavone	*P. clematidea*	*E. coli*, *P. aeruginosa*	[36]
3⁣′,4⁣′,7-Trihydroxyflavone	*R. verniciflua*	*E. coli*	[37]
Licoflavone C	*R. raetam*	*E. coli*, *P. aeruginosa*	[38]
5-Hydroxy-3,7-dimethoxyflavone-4⁣′-*O*-*β*-glucopyranoside	*C. procera*	*E. coli*, *P. aeruginosa*, *K. pneumoniae*, *S. enteritidis*	[39]
5,7,3⁣′,4⁣′-Tetrahydroxyflavone	*A. obesum*	*E. coli*, *P. aeruginosa*, *P. vulgaris*	[40]
3,5,7,3⁣′,4⁣′,5⁣′-Hexahydroxyflavone			
Vignafuran	*S. parviflorus*	*P. aeruginosa*, *E. coli*, *S. typhimurium*	[41]
3,4⁣′,5-Trihydroxy-3⁣′,7-dimethoxyflavone	*D. angustifolia*	*E. coli*	[42]
Oraristatinoside A	*O. aristatus*	*E. coli*, *P. aeruginosa*, *S. enterica*	[43]
Bropapyriferol	*B. papyrifera*	*Aggregatibacter actinomycetemcomitans*, *Fusobacterium nucleatum*, *Porphyromonas gingivalis*	[44]
5,7,3⁣′,4⁣′-Tetrahydroxy-3-methoxy-8,5⁣′-diprenylflavone			
Abyssinone VII			
Genkwanin	*C. erythrophyllum*	*E. coli*, *P. aeruginosa* *S. typhimurium*, *Shigella sonnei*, *V. cholerae*	[16]
5-Hydroxy-7,4-dimethoxyflavone			
Diosmetine	*P. campanulatus*	*E. coli*, *E. cloacae*, *K. pneumoniae*, *P. aeruginosa*	[34]
Pseudarflavone A	*P. hookeri*	*E. coli*, *P. aeruginosa*, *K. pneumoniae*	[27]
3⁣′,4⁣′,7-Trihydroxyflavone	*M. fragrans*	*E. coli*, *E. aerogenes*, *K. pneumoniae* *P. stuartii* and *P. aeruginosa*	[45]
Clerodendronone 1a	*C. formicarum*	*S. flexneri*, *P. aeruginosa*, *K. pneumoniae*	[46]
Clerodendronona 1b			
5,7-Dihydroxy-4⁣′-methoxy-flavone			
3,5,7,4⁣′-Tetrahydroxy-flavanone	*P. integerrima*	*E. coli*	[47]
Naringenin			
3,5,4⁣′-Trihidroxy,7-metoxi-flavanone			
Sakuranetin			
Vernoguinoflavone	*V. guineensis*	*E. coli*, *S. Muenchen*, *S. typhimurium*	[35]
Gliricidin 7-*O*-hexoside	*A. nidus*	*P. mirabilis*, *P. vulgaris*, *P. aeruginosa*	[48]
Norwogonin	*S. baicalensis*	*Acinetobacter baumannii*	[49]
4¢-Methoxy-5,7-dihydroxyflavone 6-C-glucoside (isocytisoside)	*A. vulgaris*	*E. coli*, *P. mirabilis*, *E. cloacae*, *K. pneumoniae*, *P. aeruginosa*	[50]
5-Methoxy-6,7-methylenedioxy-4-O-2⁣′-cycloflavan	*I. tenuifolia*	*Mycobacterium vaccae*	[51]
5,7,2⁣′,3⁣′-Tetrahydroxyflavanone			
5,2⁣′,3⁣′-Trihydroxy-6,7-methylenedioxyflavanone			
((2S)-5-Carboxymethyl-4⁣′,7-dihydroxyflavonone, 5-carbomethoxymethyl-4⁣′,7-dihydroxyflavone)	*Selaginella moellendorffii*	*E. coli*, *Helicobacter pylori*	[52]
Luteolin 7-O-*β*-D-glucoside	*C. elmaliensis*	*E. coli*, *P. aeruginosa*, *S. typhimurium*, *K. pneumoniae*	[53]
4⁣′,5-Dihydroxy-6,7-(2,2-dimethylchromeno)-2⁣′-methoxy-8-*γ,γ*-dimethylallylflavone	*A. anisophyllus*, *A. lowii*	*E. coli*, *Pseudomonas putida*	[21]
Artocarpin			
5-Hydroxy-6,7,8,2⁣′,4⁣′-pentamethoxyflavone	*A. kulbadica*	*E. coli*, *P. aeruginosa*, *S. typhi*	[54]
Apigenin	*C. scapigera*	*E. coli*, *P. aeruginosa*	[55]

**Table 4:** Antibacterial activity of flavonols.

**Flavonoid name**	**Isolated from**	**Antibacterial effect against**	**References**
3-*O*-Methylquercetin	*I. viscosa*	*S. typhimurium*	[56]
3,3⁣′-Di-*O*-methylquercetin			
Morin	*E. umbellata*	*E. coli, S. typhi*, *K. pneumoniae*, *P. aeruginosa*, *Proteus mirabilis*	[57]
3-*O*-Methylgalangin	*Heliotropium*	*E. coli*, *K. pneumoniae*, *Proteus mirabilis*, *E. cloacae*	[24]
Kaempferol-3-*O-α*-L-rhamnopyranoside	*A. chinensis*	*E. coli*	[58]
Quercetin-3-*O-α*-L-rhamnopyranoside			
Quercetin	*Buddleja salviifolia*, *M. conglomerata*, *Vernonia guineensis*, *Penstemon campanulatus*, *Indigofera aspalathoides*	*P. aeruginosa*, *E. coli*, *K. pneumoniae*, *Salmonella Muenchen*, *E. cloacae*	[34, 35, 73–75]
Quercitrin	*G. axillaris*, *E. abyssinica*	*P. aeruginosa*, *E. coli*, *S. typhimurium*	[64, 65]
Kaempferol	*V. laurentii*, *A. maritima*	*P. aeruginosa*, *E. coli*, *S. dysenteriae*, *P. vulgaris*, *P. mirabilis*, *K. pneumoniae*, *S. typhi*, *M. morganii*, *C. freundii*, *E. cloacae*	[60, 66]
Isorhamnetin-3-*O*-*α*-L-glucopyranoside	*Diplotaxis virgata*, *Diplotaxis erucoides*	*P. aeruginosa*, *Salmonella enteritidis*, *E. coli*, *A. hydrophila*, *K. pneumoniae*	[59]
Rhamnetin-3,3⁣′-di-*O-β*-D-glucopyranoside			
Quercetin-7-*O*-methylether	*R. beddomei*	*P. aeruginosa*, *E. coli*	[32]
Quercetin-3⁣′-*O-β*-D-glucopyranoside	*H. oblongifolium*	*S. typhi*, *E. coli*, *P. aeruginosa*	[61]
Quercetin-3-*O*-*α*-L-rhamnopyranoside-2⁣^″^-gallate	*S. leucantha*	*E. coli*, *P. aeruginosa*	[62]
Quercetin 3⁣′7 di-*O* methyl 3-sulfate	*A. speciosa*	*K. pneumoniae*, *E. coli*	[63]
Kaempferol 7-*O* methyl 3-sulfate			
Quercetin-3-*O-β*-D-glucosyl (1 → 4)-*α*-l-rhamnoside	*E. abyssinica*	*P. aeruginosa*, *E. coli*, *S. typhimurium*	[64]
Quercetin-5,3-dimethylether	*C. erythrophyllum*	*E. coli*, *K. pneumoniae*, *P. aeruginosa* *S. typhimurium*, *Shigella sonnei*, *V. cholerae*	[16]
Rhamnazin			
Rhamnocitrin		*E. coli*, *P. aeruginosa*, *V. cholerae*, *S. sonnei*	
Quercetin 3-methyl ether	*A. maritima*	*E. coli*, *P. aeruginosa*	[66]
Isorhamnetin-3-*O*-robinobioside			
Quercetin-3-*O*-rutinoside			
Isorhamnetin 3-*O-β-*D-glucoside	*S. glaucus*	*E. coli*	[67]
Isorhamnetin 3-*O-β-*D-rutinoside			
Kaempferol 3-*O*-[3-*O*-acetyl-6-*O*-(E)-p-coumaroyl]-b-d-glucopyranoside	*S. hymettia*	*P. aeruginosa*, *E. coli*, *E. cloacae*, *K. pneumoniae*	[68]
Astragalin (kaempferol 3-*O-β*-D-glucoside)			
Kaempferol-3-*O*-rutinoside	*C. procera*	*E. coli*, *P. aeruginosa*, *K. pneumoniae*, *S. enteritidis*	[39]
Isorhamnetin-3-*O*-rutinoside			
Quercetin-3-*O*-rutinoside			
Quercetin-3-*O-β*-D-glucopyranoside	*M. buchananii*	*V. cholerae*, *S. flexneri*	[69]
Quercetin-3-*O*-glucoside	*A. polyacantha*	*E. coli*	[70]
Quercetin 7-*O*-rutinoside	*A. nidus*	*P. mirabilis*, *P. vulgaris*, *P. aeruginosa*	[48]
Isorhamnetin 3-*O-β*-rutinoside	*G. brunneum*	*E. coli*, *K. pneumoniae*	[71]
Quercetin 3-*O-β*-rutinoside			
Quercetin-3-*O-β-*D-galactopyranoside	*C. stellata*	*E. coli*, *P. aeruginosa*	[72]
Macaragin	*M. conglomerata*	*E. coli*, *P. aeruginosa*, *K. pneumoniae*	[74]
Myricetin-3-*O*-(3⁣^″^-*O*-methyl)-*α-*L-rhamnopyranoside	*M. hexandra*	*P. aeruginosa*, *E. coli*, *S. typhimurium*, *K. pneumoniae*	[76]
Pollenitin-3-*O-β*-D-mannopyranoside (ephedroside B)	*E. sinica*	*P. aeruginosa*	[77]
Rutin	*R. chalepensis*	*E. coli*, *P. aeruginosa*	[78]
Rutin 3-methyl ether			
6-Hydroxy-rutin 3,7-dimethyl ether			
Tiliroside	*C. elmaliensis*	*E. coli*, *P. aeruginosa*, *S. typhimurium*, and *K. pneumoniae*	[53]
Lanneaflavonol	*L. alata*	*P. aeruginosa*	[79]
Fisetin	*R. verniciflua*	*E. coli*	[37]
Fustin			
Ericoside	*Erica mannii*	*E. coli*, *K. pneumoniae*, *P. stuartii*	[80]

**Table 5:** Antibacterial activity of isoflavonoids.

**Flavonoid name**	**Isolated from**	**Antibacterial effect against**	**References**
7,4⁣′-Dihydroxy-5,3⁣′-dimethoxyisoflavone	*H. oblongifolium*	*S. typhi*, *E. coli*, *P. aeruginosa*	[61]
Genistin	*F. strobilifera*	*P. aeruginosa*, *E. coli*	[26]
5,7-Dihydroxy-20-methoxy-30,40-methylenedioxyisoflavanone	*U. picta*	*E. coli*, *P. vulgaris*	[81]
5,7,4⁣′-Trihydroxy-2⁣′,3⁣′-dimethoxyisoflavanone			
40,5-Dihydroxy-20,30-dimethoxy-7-(5-hydroxyoxychromen-7yl)-isoflavanone			
4⁣′,5,7-Trihydroxy-2⁣′-methoxyisoflavanone (isoferreirin)			
2⁣′,4⁣′,5,7-Tetrahydroxy-6-(3-methylbut-2-enyl)isoflavanone			
2⁣′,4⁣′,5,7-Tetrahydroxyisoflavanone			
Alpinumisoflavone	*F. chlamydocarpa*	*E. cloacae*, *M. morganii*, *Proteus mirabilis*	[19]
Genistein	*S. parviflorus*, *F. chlamydocarpa*	*E. cloacae*, *M. morganii*, *P. aeruginosa*, *Serratia marcescens*	[19, 41]
Laburnetin	*F. chlamydocarpa*	*C. freundii*, *E. cloacae*, *M. morganii*, *P. mirabilis*, *S. typhi*	[19]
Gancaonin Q	*D. angusticornis*	*C. freundii*, *E. aerogenes*, *K. pneumoniae*, *M. morganii*, *P. mirabilis*, *P. aeruginosa*, *S. dysenteriae*, *S. flexneri*, *S. typhi*	[82]
Stipulin		*C. freundii*, *K. pneumoniae*, *P. mirabilis*, *P. vulgaris*, *P. aeruginosa*, *S. dysenteriae*, *S. flexneri*, *S. typhi*, *Salmonella typhimurium*	
Angusticornin B		*C. freundii*, *E. aerogenes*, *E. cloacae*, *E. coli*, *K. pneumoniae*, *M. morganii*, *P. mirabilis*, *P. vulgaris*, *P. aeruginosa*, *S. dysenteriae*, *S. flexneri*, *S. typhi*, *S. typhimurium*	
Bartericin A			
Abyssinone V 4⁣′-*O*-methyl ether	*E. caffra*	*E. coli*, *K. pneumoniae*	[83]
6,8-Diprenylgenistein			
Alpinumisoflavone			
Burtinnone			
Millexatin A	*M. extensa*	*S. typhimurium*, *P. aeruginosa*	[84]
Millexatin F			
3⁣′-Methylorobol			
Auriculatin			
Scandenone			
Elongatin			
Auriculasin			
2⁣′-Deoxyisoauriculatin			
Isoauriculatin			
7,3-Dihydroxy-8,4⁣′-dimethoxyisoflavone	*S. parviflorus*	*P. aeruginosa*, *S. marcescens*	[41]
Derrone	*R. raetam*	*E. coli*, *P. aeruginosa*	[38]
Lachnoisoflavone A	*C. lachnophora*	*E. coli*, *K. pneumoniae*	[85]
Lachnoisoflavone B			
Wighteone	*E. lysistemon*	*E. coli*	[86]

**Table 6:** Antibacterial activity of flavanols.

**Flavonoid name**	**Isolated from**	**Antibacterial effect against**	**References**
Piafzelechin	*F. cordata*	*E. coli*, *S. dysenteriae, P. mirabilis*, *K. pneumoniae*, *P. aeruginosa*, *S. typhi*, *M. morganii*, *C. freundii*, *E. cloacae*	[19]
(–)-Epicatequina(2-(3,4-di-hidroxifenil)-3,4-di-hidro-2H-cromeno-3,5,7-triol)	*M. indica*	*E. coli*, *Azospirillum lipoferum*	[31]
Catechin	*S. latifolia*	*E. coli*, *K. pneumoniae*, *P. aeruginosa*	[87]
Epicatechin	*S. latifolia*, *E. crispa*, *E. schimperi*, *G. axillaris*, *S. kamerunensis*, *M. buchananii*, *A. polyacantha*	*E. coli*, *K. pneumoniae*, *P. aeruginosa*, *S. dysenteriae*, *E. cloacae*, *Klebsiella oxytoca*, *P. vulgaris*, *P. mirabilis*, *V. cholerae*, *S. flexneri*, *E. aerogenes*	[65, 69, 70, 87–89]
Flavan derivative	*E. schimperi*	*K. pneumoniae*, *E. coli*, *S. dysenteriae*	[90]
Epigallocatechin-3-gallate	*E. livingstoniana*	*Stenotrophomonas maltophilia*	[91]

**Table 7:** Antibacterial activity of flavanonols and tetraflavonoids.

**Flavonoid name**	**Isolated from**	**Antibacterial effect against**	**References**
3-*O*-Ethyl-dihydroquercetin	*M. hexandra*	*P. aeruginosa*, *E. coli*, *S. typhimurium*, *K. pneumoniae*	[76]
Dihydroflavonol	*T. nilotica*	*E. coli*, *S. typhi*	[92]
Taxifolin 3-*O-α*-L-rhamnopyranoside	*E. mannii*	*E. coli*, *E. aerogenes*, *K. pneumoniae*, *P. stuartii*	[80]
Lemairone A	*Z. lemairei*	*E. coli*, *E. aerogenes*, *K. pneumoniae*, *P. stuartii*	[93]

## Data Availability

The corresponding author has made all of the study's data available upon request. They are all included in this manuscript.

## Supporting Information

Supporting Information Additional supporting information can be found online in the Supporting Information section. Table S1. Antibacterial activity of flavonoids isolated from plants against Gram-negative bacteria (2004–2025).
