# Supplementary material for: Flavonoids: A Review of Antibacterial Activity Against Gram-Negative Bacteria
Source: Int J Microbiol. 2025 Sep 15;2025:9961121. doi: 10.1155/ijm/9961121 (PMC12453933; doi:10.1155/ijm/9961121)
Supplement: Supporting Information — Additional supporting information can be found online in the Supporting Information section. Table S1. Antibacterial activity of flavonoids isolated from plants against Gram-negative bacteria (2004–2025). [file 9961121.f1.zip › IJM_supplementary material.docx]

**Table 1.** Antibacterial activity of flavonoids isolated from plants against Gram-negative bacteria (2004 to 2025).

| **Flavonoid isolate** | **Method of isolation and**  **identification** | **Species and plant part** | **Bacteria tested** | **Reference** |
| --- | --- | --- | --- | --- |
| 6-methoxy,7-methyl-luteolin,  Hispidulin,  Hispidulin 7-*O-β*-d-lucuronopyranoside,  Apigenin-7-*O-β*-d-glucuronopyranoside,  Apigenin-7-*O*-(6-methoxy)-*β*-d-glucuronopyranoside | 1D and 2D NMR, HR-ESI-MS | Aerial parts of *C. apungens* | *P. aeruginosa, Pseudomonas fragi* | [29] |
| 3,3′-di-*O*-methylquercetin,  3-*O*-methylquercetin | CC, TCL | *I. viscosa* | *E. coli, Salmonella typhimurium* | [56] |
| Morin (2-(2,4-Dihydroxyphenyl)-3,5,7-trihydroxychromen-4-one) | CC, EIMS, ^1^H-NMR, ^13^C-NMR | Berries of *E. umbellata* (Thunb.). | *E. coli, S. typhi, K. pneumonia, P. aeruginosa and P. mirabilis* | [57] |
| Chrysoeriol-7-*O-β-D*-xyloside,  Luteolin-7-*O-β-D*-apiofuranosyl-(1→2)-*β-D*-xylopyranoside,  Chrysoeriol-7-*O-β-D*-apiofuranosyl-(1→2)-*β-D*-xylopyranoside,  Chrysoeriol-7-*O-α-L*-rhamnopyranosyl-(1→6)-*β-D*-(4"-hydrogeno sulfate) glucopyranoside,  Isorhamnetin-3-*O-α-L*-rhamnopyranosyl-(1→6)-*β-D*-glucopyranoside | 1D (^1^H and ^13^C-NMR), 2D, CC, HR-TOF-ESI-MS | Aerial parts of *G. glandulosum* Turrill | *V. cholerae* NB2, PC2, SG24 (1) and CO6 | [30] |
| (–)-epicatequina-3-*O-β*-glicopiranosídeo,  5-hidroxi-3-(4-hidroxilfenil)pirano[3,2-g]cromeno-4(8H)-ona,  6-(p-hidroxibenzil) taxifolin-7-*O-β-D*-glicoside (tricúspide),  quercetina-3-*O-α*-glicopiranosil-(1→2)-β-glicopiranosídeo,  (–)-epicatequina(2-(3,4-di-hidroxifenil)-3,4-di-hidro-2H-cromeno-3,5,7-triol) | Not detailed | Leaves of *M. indica* L. | *E. coli, Azospirilium lipoferum* | [31] |
| 3-*O*-methylgalangin,  3,7-*O*-dimethylgalangin,  Naringenin,  Pinocembrin,  7-*O*-methyleriodictyol,  3-*O*-methylsorhamnetin | TLC, HPLC-UV | Resinous exudates of *Heliotropium filifolium* (Miers) I.M. Johnst., *H. huascoense*, *H. sinuatum* I.M. Johnst. | *E. cloacae, E. coli, K. pneumoniae, P. mirabilis* | [24] |
| 2’,4’-dihydroxychalcone | CC, ^1^H and ^13^C NMR, HPLC | Aerial parts of *F. oolepis* S. F. Blake | *E. coli* (ATCC 25922)*, P. aeruginosa* (ATCC 27853) | [23] |
| Isorhamnetin-3-*O-α*-Lglucopyranoside,  Rhamnetin-3,3ʹ-di-*O-β*-D-glucopyranoside | 1D and 2D NMR, UV and MS | *D. virgata* D.C. and *D. Erucoides* (L.) D.C. | *P. aeruginosa* ATCC 9027, *Salmonella enteritidis* ATCC 14028, *E. coli* ATCC 25922, *A. hydrophila* ATCC 1943, *K. pneumoniae* ATCC 13833 | [59] |
| 5,7-dihydroxy-4′-methoxyisoflavone,  Quercetin-7-*O*-methylether,  C-glycosides,  Isovitexin,  5,7,3′,4′-tetrahydroxy-6-C-*β*-D-glucopyranosyl flavone | ^1^H and ^13^C NMR | Flowers of *R. beddomei* Baker | *P. aeruginosa, E. coli* | [32] |
| Apigenin,  Kaempferol,  Quercetin,  Eriodictyol,  Taxifoliz,  Naringenin | 1D and 2D  NMR, HPLC-DAD-MS, CD, IR | Aerial parts of *O. dictamnus* | *P. aeruginosa, K. pneumoniae* | [28] |
| 7,4'-di-hidroxi-5,3'-dimetoxiisoflavona,  Quercetina-3'-*O-ß*-D-glucopiranosídeo | CC, HP-TLC,  ^1^H and ^13^C NMR | Whole plant *H. oblongifolium* | *Salomella typhi* 19 18 17 20 21 25 *E. coli* 22 24 23 25 26 30 *P. aeruginosa* | [61] |
| 8, 3'-diprenyl 5, 7, 4'-trihydroxy flavanone,  Genistin | CC, UV, IR, 2D NMR and MS | Roots of *F. strobilifera* | *P. aeruginosa* ATCC 7853*, E. coli* ATCC 25922 | [26] |
| Quercetin | Not detailed | *I. aspalathoides* | *E. coli, P. aeruginosa* | [75] |
| Alpinumisoflavone,  Genistein,  Laburnetin,  Luteolin,  Catechin,  Epiafzelechin | 2D NMR, COSY, HMQC, HMBC | Stem bark of *F. cordata* Thunberg | *E. coli* (LMP0101U), *Shigella dysenteriae*  (LMP0208U), *P. mirabilis* (LMP0504G), *K. pneumoniae* (LMP0210U), *P. aeruginosa* (LMP0102U), *Salmonella typhi* (LMP0209U), *Morganella morganii* (LMP0904G), *Citrobacter freundii* (LMP0904G), *E. cloacae* (LMP1104G) | [105] |
| Gancaonin Q,  Stipulin,  Angusticornin B,  Bartericin A | UV, ^1^H and ^13^C NMR | Twigs of *D. angusticornis* | *E. coli* LMP0101U, *S. dysenteriae* LMP0208U, *P. vulgaris* LMP0103U, *P. mirabilis* LMP0504G, *Shigella flexneri* LMP0313U, *K. pneumoniae* LMP0210U, *P. aeruginosa* LMP0102U, *S. typhi* LMP0209U, *S. typhimurium* LMP0704G, *M. morganii* LMP0904G, *Enterobacter aerogens* LMP1004G, *Citrobacter freundii* LMP0804, *E. cloacae* LMP1104G | [82] |
| 4,2',4'-trihydroxychalcone,  4,2',4'-trihydroxy-3-prenylchalcone | 1 D 2D NMR,  ^1^H and ^13^C NMR | Twigs of *T. obovoidea* N.E. Brown | *E. coli LMP0101U, Shigella dysenteriae LMP0208U,*  *P. vulgaris LMP0103U, P. mirabilis LMP0504G,*  *S. flexneri LMP0313U, K. pneumoniae LMP0210U,*  *P. aeruginosa LMP0102U, S. typhi LMP0209U,*  *M. morganii LMP0904G, E. aerogenes LMP1004G,*  *Citrobacter freundii LMP0904G, E. cloacae LMP1104G* | [18] |
| Isobavachalcone,  Stipulin,  4-hydroxylonchocarpin,  Kanzonol C,  Amentoflavone | TLC, HPLC, UV-Vis, NMR, and MS | Twigs of *D. barteri* Bureau var. Multiradi- ata | *S. typhi, S. dysenteriae, S. flexneri, E. coli, E. aerogens, E. cloacae, P. mirabilis,* *P. vulgaris,* *P. aeruginosa, K. pneumoniae, M. morganii, Citrobacter freundii* | [19] |
| Chrysoeriol | HPLC-DAD | Whole fruit, peel and seed of *C. frutescens* | *E. coli, P. aeruginosa, K. pneumoniae* | [33] |
| Quercetin-3-*O- α*-L- rhamnopyranoside -2’’- gallate | UV, ^1^H and ^13^C NMR | Flores de *S. leucanta* | *E. coli*, *P. aeruginosa* | [62] |
| Epicatechin,  Catechin | CC, VLCUV, MS, IR, ^1^H and ^13^C NMR (2D) | Stem of *S.latifolia* Jacq | *E. coli* e *P. aeruginosa* | [87] |
| Epicatechin,  Catechin,  Gallocatechin,  Quercitrin,  Hyperoside | P-TLC, UV,  GC-MS, ^1^H | Leaf *Euclea crispa* subsp. | *A. baumannii, E. coli, Haemophilus influenzae, K. pneumoniae, Moraxella catarrhalis, P. aeruginosa* | [88] |
| 5.7.4’-trimethoxyflavone | Was performed according to Maia *et al.* (2011). | Aerial parts of *P. clematidea* | *P. aeruginosa -* P03*, P. aeruginosa - ATCC 25853, E. coli - ATCC 25922, E. coli – 5, S. enterica* ATCC 6017, *S. enterica* LM08, *S. sonnei* | [40] |
| Myricetin 3-*O* (3′′ -*O*-methyl) *α*-L-rhamnopyranoside,  3-*O*-ethyl-dihydroquercetin (3-*O*-ethyltaxifolin) | 1D and 2D NMR, MS,  TLC, UHPLC-MS | Leaves and fruits of *M. hexandra* (Roxb.) Dubard | *P. aeruginosa* RCMB 010043, *E.coli* RCMB 010052, *Salmonlla typhimurium* RCMB 010072, *K. pneumonia* RCMB 0100223-5 | [76] |
| Abyssione-V 4′-*O*-methyl ether,  6,8-diprenylgenistein,  Alpinumisoflavonone,  Burtinnone | CC, TLC, ^1^H and ^13^C NMR | Stem bark of *E. caffra* Thunb. | *E. coli, K. pneumonia* | [83] |
| Macaragin,  Quercetin | UV,  ^1^H and ^13^C NMR and HSQC | Leaves and stem bark of *M. conglomerata* | *E. coli* ATCC 25922, *P. aeruginosa* ATCC 27853, *K. pneumoniae* ATCC 31488 | [74] |
| Kaempferol | ^1^H and ^13^C NMR | Leaves, twigs and the roots of *V. laurentii* De Wild | *E. coli* LMP0101U, *S. dysenteriae* LMP0208U, *P. vulgaris* LMP0103U, *P. mirabilis* MP0504G, *Shigella flexneri* LMP0313U, *K. pneumoniae* LMP0210U, *P. aeruginosa* LMP0102U, *S. typhi* LMP0209U, *Morganella morganii* LMP0904G, *Enterobacter aerogens* LMP 1004G, *Citrobacter freundii* LMP0904G,  *E. cloacae* LMP1104G | [60] |
| Pollenitin-3-*O-β-*D-mannopyranoside (ephedroside B),  Kaempferol-3-*O-α*-L-rhamnopyranoside,  Gossypetin- 8-*O-β*-D-glucopyranoside,  Apigenin-8-C-*β*-D-glucopyranosid,  Pollenin B,  Herbacetin -3-*O-α*-Lrhamnopyranoside- 8-*O-β*-D-glucopyranoside,  Kaempferol-7- *O-β-*D-glucopyranoside, isoscutellarein-3-*O-α*-L-rhamnopyranoside | NMR, HR-ESI-TOF-MS and IR | Herbaceous stems of *E. asinica* Stapf. | *P. aeruginosa,*  *E. coli* | [77] |
| Quercetin 3-methyl ether,  Kaempferol,  Vitexin,  Isovitexin,  Isorhamnetin-3-*O*-robinobioside,  Quercetin-3-*O*-rutinoside | UV, IR, NMR  and ES-MS, TCL, HPLC | Aerial parts and roots *A. Maritima* (Mart.) St. Hil. | *E. coli* (ATCC 10538 and Ec 26.1)  *P. aeruginosa* (ATCC 27853 and 290D) | [66] |
| Oleanan-12-ene-2a,3b –diol,  Epicatechin | ^1^H and ^13^C RMN, HR-ESI-MS | Stem bark of *S. kamerunensis* | *E. cloacae, Klebsiella oxytoca, P. aeruginosa,*  *P. vulgaris, E. coli, P. mirabilis, k. pneumonia* | [90] |
| Rutin,  Rutin 3-methyl ether,  6-hydroxy-rutin 3,7-dimethyl ether | ^1^H and ^13^C NMR, DEPTQ, COSY, HSQC and HMBC | Leaves, stem, fruits and roots of *R. chalepensis* L. | *E. coli* (NCTC 12241), *P. aeruginosa* (NCTC 12903) | [78] |
| 5,7,3′,4′-tetrahydroxy flavone,  3,5,7,3′,4′,5′-hexahydroxyflavone | ^1^H and ^13^C NMR, DEPT 90 and 135, COSY, HMBC and MS | Stem of *A. obesum* | *E. coli, P. aeruginosa* e *P. vulgaris* | [39] |
| Khonklonginol A,  Lupinifolinol,  Flemichin D,  Lupinifolin,  20 -hydroxylupinifolinol,  3,5,20,40-tetrahydroxy-600,600-dimethylpyrano(200,300:7,6)-8- (3000,3000-dimethylallyl)flavone,  (2R,3R)-3,5-dihidroxi-4'-metoxi-6',6'-dimetilpirano(2',3':7,6)-8-(2'',3''-epóxi-3''-metilbutil)flavanona,  (2R,3R)-3,5,2',4'-tetrahidroxi-6',6'-dimetilpirano(2',3':7,6)-8-(3',3'-dimetil-2'-buteno)flavanona,  Tectorigenin,  Kaempferol,  Kaempferol-7-O-b-D -glucopyranoside,  Genistein-7-O-b-D -glucopyranoside,  Genistin,  Astragalin | CC, TLC, ^1^H and ^13^C NMR, R-APCI-MS | Roots of *E. chinense* | *E. coli* (ATCC 25922), *K. pneumoniae* (DMST 8216), *P. aeruginosa* (ATCC 27853) | [106] |
| Pseudarflavone A,  Pseudarflavone B,  6,7-(2″,2″-dimethylchromano) flavanone,  6-prenylpinocembrin,  Hiravanone,  6-prenyl-3′-methoxyeriodictyol,  Boeravinone L,  Desmoxyphyllin A,  Orobol,  6-prenylpinocembrin acetate,  7-benzyloxy-6-prenylpinocembrin | ^1^H and ^13^C NMR and IR | Whole plant *P. hookeri* Wight& Arn. | *E. coli* ATCC11775, *P. aeruginosa* ATCC27853, *K. pneumoniae* ATCC12296 | [27] |
| Lanneaflavonol,  Dihydrolanneaflavonol,  Myricetin-3-*O*-α-L-rhamnopyranoside (myricitrin),  Myricetin-3-*O*-α-L-arabinofuranoside(betmidin) | IR, UV and ^1^H NMR | Stem and roots of *L. Alata* (Engl.) Engl | *E. coli* ATCC 25922 and ATCC 35218, *P. aeruginosa* ATCC 27853 and 35032, *K. pneumoniae* ATCC 700603 | [79] |
| Colucins A and B,  Colucone | TLC, UV, IR,  MS and 2D-NMR | Whole plant *C. armata* | *E. coli* ATCC 25922, *P. aeruginosa* ATCC 10145, *Pseudomonas pseudomallei* ATCC 23343, *Salmonella typhi* ATCC 10749) | [20] |
| Fustin,  3,4,7-trihydroxyflavone,  Fisetin | TLC, ^1^H and ^13^C NMR, UV | Heartwoods *R. verniciflua* | *E. coli* (KTCT 1924), *S. typhimurium* (KTCT 1925) | [36] |
| Ericoside,  Taxifolin 3-*O-α*-L-rhamnopyranoside | MS, RMN, CC | Whole plant of *E. mannii* | *E. coli* (ATCC8739, ATCC10536, AG100, AG102 and AG100ATet), *Enterobacter*  *aerogenes* (ECCI69), *K. pneumoniae* (KP55 and ATCC11296), *Providencia stuartii* (ATCC29916 and NAE16) | [80] |
| Lemairones A and B | MS, IR, UV, 1D and 2D NMR, HPCL | Leaves of *Z. lemairei* | *E. coli* (ATCC8739 and ATCC10536, AG100, AG102 and AG100Atet), *E. aerogenes* (ECCI69), *K. pneumoniae* (KP55 and ATCC11296) *P. stuartii* (ATCC29916 and NAE16). | [93] |
| Lycoflavone C,  Derrone | ^1^H and ^13^C NMR, HR-EIMS | Flores de *R. raetam* | *E. coli (ATCC 25922), P. aeruginosa (ATCC 27950)* | [37] |
| Kaempferol-3-*O*-rutinoside,  Isorhamnetin-3-*O*-rutinoside,  Quercetin-3-Orutinoside,  5-hydroxy-3,7-dimethoxyflavone-40-*O-*β-glucopyranoside | UV, IR, ^1^H and ^13^C NMR, DEPT, 2D (1)H-(1)H COSY, HSQC, HMBC and NOESY | *C. procera* Ait. | *E. coli, P. aeruginosa*  *K. pneumoniae*  *S. enteritidis* | [38] |
| Quercetin-3-*O-α*-L-arabinopyranoside 1, Epicatechin,  Quercetin,  Kaempferol-3-*O-α*-L-rhamnopyranoside, Quercetin-3-*O-α*-L-rhamnopyranoside, Quercetin-3-*O-β*-D-glucopyranoside, Epigallocatechin 7,  Kaempferol,  Quercetin-3-*O*-[*α*-L-rhamnopyranosyl-(1→6)-*β*-D-glucopyranoside] | 1D and 2D 1Hand13C NMR spectrawere, UV | Leaves of *M. buchananii* | *V. cholerae* (O1 and O139), *V. cholerae* (non-O1, non-O139 (strains CO6 and PC2), *S. flexneri* | [69] |
| Epicatechin,  Quercetin-3-*O*-glucoside | CC | Leaves of *A. polyacantha* Willd. | *E. coli* (ATCC8739, ATCC10536, AG102, and AG100Atet), *E. aerogenes* (ATCC13048, CM64, EA27 and EA289), *K. pneumoniae* (ATCC11296, KP55 and KP63), *P. stuartii* (ATCC29916 and NEA16), *P. aeruginosa* (PA01 and PA124) | [70] |
| Isorhamentin 3-*O-β*-D-glucoside,  Isorhamentin 3-*O-β*-D-rutinoside | 1D and 2D-NMR, UV, IR, HR-MS | Leaves of *S. glaucus* L. | *E. coli,*  *P.aeruginosa* | [67] |
| Dorsmanin A, B, C, D, E, F, G, I,  6,8-diprenyleriodictyol | Not detailed | Twigs of *D. mannii* | *P. stuartii, P. aeruginosa, K. pneumoniae, E. aerogenes, E. coli* | [25] |
| 3,5,7,3′,4′-pentahydroxyflavan (epicatechin), Flavan derivative | ^1^H and ^13^C NMR, DEPT-135, HMBC, HSQC, COSY, IR, UV-Vis | Stem Barks of *E. schimperi* | *K. pneumoniae, E. coli, P. mirabilis* | [89] |
| Luteolin 4’-neohesperidoside | ^1^H and ^13^C NMR,  CC, UV | Frutas de *P. Emblica* L. | *K. pneumoniae* , fosA-positive shiga toxin producing the *E. coli* serogroup O111 (STEC O111), | [17] |
| 3′,4′,7-trihydroxyflavone | ^1^H and ^13^C NMR | Seeds of *M. fragrans* Houtt. | *E. coli, E. aerogenes, K. pneumoniae,*  *P. stuartii, P. aeruginosa* | [45] |
| Vernoguinoflavone,  Luteolin,  Quercetin | LC-MS, 1D and 2D NMR, HR-ESI-MS | Aerial parts of *V. guineensis* Benth | *E. coli* (ATCC 25922), *Shigella flexineri* (NR 518), *S. Muenchen, S. typhimurium, S. typhi* (ATCC 19430) | [34] |
| 5,7-dihydroxy-40-α-D-glucopyranosylflavone (clerodendronone1a),  5,7-dihydroxy-40-β-D–glucopyranosylflavone (clerodendronone 1b),  5,7-dihydroxy-40-methoxy-flavone | CC, TLC, LC-MS-QTOF, ^1^H and ^13^C NMR | Leaves of *C. formicarum* G€urke | *K. pneumonia* NR41916 e ATCC13883, *Shigella flexineri* NR518, *Pneudmonas enteric* NR13555 | [46] |
| 3,5,7,4^/^-tetrahydroxy-flavanone,  Naringenin,  3,5,4^/^-trihydroxy,7-methoxy-flavanone,  Sakuranetin | NMR e MS | Galls of *P. integerrima* | *E. coli, K. pneumonia.* | [47] |
| Gliricidin7-*O*-hexoside,  Quercetin7-*O*-rutinoside | GC/MS | Whole plant *A. nidus* fern | *P. mirabilis, P. vulgaris P. aeruginosa* | [48] |
| Kaempferol 3-*O*-[3-*O*-acetyl-6-*O*-(E)-*p*-coumaroyl]-β-D-glucopyranoside,  Kaempferol 3-*O-*β-Dglucopyranoside (astragallin) | ^1^H and ^13^C NMR | Flowering plants of *S. hymettia* Boiss. Et Spruner | *P. aeruginosa* (ATCC 27853), *E. coli* (ATCC 25922), *E. cloacae* (ATCC 13047), *K. pneumoniae* (ATCC 13883) | [68] |
| Norwogonin (5,6,7-trihydroxyflavone) | MS, UV, ^1^H and ^13^C NMR, HPLC | *S. baicalensis* | *A. baumannii* | [49] |
| Quercetin 3'7 di-*O* methyl 3- sulfate,  Kaempferol 7-*O* methyl 3-sulphate | Spectral studies and TLC | Root of *A. speciosa* (Burm.f) Boj. | *K. pneumoniae ATCC 10031, E. coli ATCC 10536* | [107] |
| 5-methoxy-6,7-methylenedioxy-4-*O*-2'-cycloflava,  5,7,2',3'-tetrahydroxyflavanone, 5,2',3'-trihydroxy-6,7-methylenedioxyflavanone,  5-hydroxy-6,7-dimethoxyisoflavone-2'-*O*-*β*-d-glucopyranoside,  5,2',3'-dihydroxy-6,7-dimethoxyisoflavone | 1D and 2D NMR, HR-ESI-MS. | Underground parts of *I. tenuifolia* Pall. | *E. coli* (458 B4) *P. aeruginosa* (SG137 B7) *P. aeruginosa* (K799/61 B9) | [51] |
| (2S)-5-carboxymethyl-4′,  7-dihydroxyflavonone,  5- carbomethoxymethyl-4′,  7-dihydroxyflavone | UV, 1D and 2D NMR, ECD | The whole plant *S. moellendorffii* Hieron. | *E. coli, K. pneumoniae, S. dysenteriae, H. pylori* | [52] |
| Tiliroside,  Luteolin 7-*O-β*-D-glycoside | 1D, 2D NMR, HR ESIMS | Aerial parts of *C. elmaliensis* (Hub.-Mor. & Matthews). | *E. coli* (ATCC 23999), *P. aeruginosa* (ATCC 27853), *S. typhimurium* (CCM 5445), *K. pneumoniae* (CCM 2318) | [53] |
| Dihydroflavonol | IR, UV, ^1^H NMR, MS | Leaves of *T. nilotica* | *E. coli, S. typhimurium* | [92] |
| Quercetin-3-*O*-*α-*l-rhamnoside (quercitrin), Quercetin-3-*O*-*β-D*-glucosyl (1→4)-α-l-rhamnoside | 1D and 2D NMR, HRMS | Stem bark of *E. abyssinica* | *P. aeruginosa* ATCC 27853, *E. coli* ATCC 25922, *S. typhimurium* ATCC 14028 | [64] |
| Vignafuran,  (6ar, 11ar)-medicarpin,  Daidzein,  Formononetine,  Genistein,  8-*O*-methylretusin,  7,30 -dihydroxy-8,40-dimethoxyisoflavone | 1 D and 2 D NMR, MS,  ^1^H NMR | Stems of *S. parviflorus* | *E. coli, S. typhymurium, P. aeruginosa, Serratia marcescens* | [40] |
| 4¢-methoxy-5,7-dihydroxyflavone 6-C-glucoside (isocytisoside) | TLC, CC, UV, NMR | Leaves and stems of *A.vulgaris* L. | *E. coli* ATCC 25922, *P. mirabilis* NCTC 6635, *E. cloaceae* ATCC 10699, *K. pneumoniae* ATCC 27736, *P. aeruginosa* ATCC 27853 | [50] |
| 5,7-dihydroxy-20-methoxy-30,40-methylenedioxyisoflavanone,  5,7,4’-trihydroxy-2’,3’-  Dimethoxyisoflavanone,  40,5-dihydroxy-20,30-dimethoxy-7-(5-  Hydroxyoxychromen-7yl)-isoflavanone,  4’,5,7-trihydroxy-2’-methoxyisoflavanone  (isoferreirin),  2’,4’,5,7-tetrahydroxy-6-(3-methylbut-2-enyl)isoflavanone,  2’,4’,5,7-tetrahydroxyisoflavanone | UV, IR, MS,  1D and 2D NMR | Roots of *U. picta* Desv. (Syn. Doodia picta Roxb | *E. coli* NCTC9001, *P. vulgaris* NCTC4175 | [81] |
| 3,4′,5-trihydroxy-3′,7-dimethoxyflavone | UV, MS, 1D and 2D NMR | Leaves of *D. angustifolia* | *E. coli* ATCC 25922 | [41] |
| Oraristatinoside A | 1D- and 2D NMR and HR-ESIMS | Aerial parts of *O. aristatus* | *E. coli* ATCC 25922, *P. aeruginosa* ATCC 9027, *S. enterica* ATCC13076 | [42] |
| Bropapyriferol,  5,7,3′,4′-tetrahydroxy-3-methoxy-8,5′-diprenylflavone,  Broussoflavonol b,  Broussonin a,  Isoliquiritigenin,  Broussoflavan a,  Abyssinone vii,  E3,5,7,3′,5′-pentahydroxyflavanone | 1D and 2D NMR, CC, HPLC | *B. papyrifera* | *Aggregatibacter actinomycetemcomitans*  (ATCC 43717), *Fusobacterium nucleatum*  (ATCC 10953), *Porphyromonas gingivalis* (ATCC 33277) | [43] |
| Rhamnocitrin,  Quercetin-5,3_-dimethylether,  Rhamnazin,  Genkwanin,  5-hydroxy-7,4_-dimethoxyflavone | CC and crystallization | Leaves ofde *C. erythrophyllum* | *E. coli, K. pneumoniae, P. aeruginosa, S. typhimurium*  *Shigella sonei, V. cholerae* | [16] |
| Quercetin-3-*O-b*-Dgalactopyranoside (hyperin) | 1D and 2D NMR, HR-MS | Whole plant *C. stellata* Cav. | *E. coli* CIP 54.127, *P. aeruginosa* ATCC 9027 | [72] |
| Quercetina | TCL, ESI-TOF-MS, 1D and 2D NMR | Leaves of *B. salviifolia* (L.) Lam. | *E. coli* e *K. pneumoniae* | [73] |
| Millexatin A,  Millexatin F,  3′-methylorobol,  18 auriculatin,  19,20 scandenone,  23 elongatin,  24 auriculasin,  25 2′-deoxyisoauriculatin,  20 isoauriculatin | ^1^H NMR  1D and 2D NMR | Stems of *M. extensa* | *S. typhimurium* TISTR781 *P. aeruginosa* TISTR 292 | [84] |
| Luteolin,  Diosmetin,  Quercetin | ^1^H and ^13^C NMR, GC–MS | Arial part of *P. campanulatus* (Cav.) | *E. coli* (ATCC 25922), *E. cloacae* (ATCC  13047), *K. pneumoniae* (ATCC 13883), *P. aeruginosa* (ATCC 227853); | [44] |
| Isorhamnetin 3-*O-β*-rutinoside,  Quercetin 3-*O-β*-rutinoside (rutin), Phlorizine | ^1^H and ^13^C NMR, HPLC, MS | Aerial parts of *G. Brunneum* | *P. aeruginosa* ATCC 27853, *E. coli* ATCC 25922 and *K. pneunomoniae* ATCC 70603 | [71] |
| Lachnoisoflavone A,  Lachnoisoflavone B | 1D and 2D NMR, HRMS | Whole plant *C. lachnophora* | *K. pneumoniae* subsp. ozaenae (DSM 681), *P. aeruginosa* (DSM 1128), *E. coli* (DSM 682) | [108] |
| 5-hydroxy-6,7,8,2’,4’-pentamethoxyflavone | 1D and 2D NMR | Arial parts *A.*  *Kulbadica* | *E. coli* PTCC 1533, *P. Aeruginosa* PTCC 1310, *S. typhi* PTCC 1609 | [54] |
| Kaempferol-3-*O-α*-L-rhamnopyranoside,  Quercetin-3-*O-α*-L-rhamnopyranoside,  Luteolin | UV, ^1^H and ^13^C NMR | Leaves of *A.*  *chinensis* | *E. coli* NRRL B-210 | [58] |
| Quercitrin,  Epicatechin | CC, MS, IR, 1D and 2D NMR | Leaves of *G. axillaris* | *E. coli* (ATCC 10536), *P. aeruginosa* (ATCC 9027) | [65] |
| 2',4'-dihydroxy-4- methoxy-3'-prenyldihydrochalcone,  4-hydroxyonchocarpin,  Isobavachalcone,  2',4'-dihydroxy-3,4-(2",2"- dimethylchromeno)-3'-prenyldihydrochalcone,  5,7-dihydroxy- 4'-methoxy-6-prenylflavanone, 5-hydroxy-6,7-(2,2- dimethylchromano)-4'-methoxyflavanone,  4',5-dihydroxy-6,7- (2,2-dimethylchromeno)-2'-methoxy-8-γ,γ-dimethylallylflavone,  Artocarpin | Not detailed | Leaves and heartwoods of *A. anisophyllus,*  *A. lowii* King | *E. coli, Pseudomonas putida* | [21] |
| Apigenin,  Crysoeriol | TLC, HPLC, UV, 1H  and 13C NMR | Roots, stem, leaves and  Inflorescences of *C. scapigera* | *E. coli* ATCC 10538*, P. aeruginosa* ATCC 27853 e 290 D | [55] |
| 4',7-dihydroxy-2 '' ,2 '' -dimethylpyrano [5 '' ,6 '' :5,6]-isoflavone,  daidzein,  Wighteone,  4',5,7- trihydroxy-6-(2 '' -hydroxy-3 '' - methylbut-3 '' - enyl)isoflavone , alpumisoflavone,  Derrone,  6,8-di-prenylgenistein,  Erysenegalensein e,  Lysistisoflavone [isoerysenegalensein e],  2',5,7-trihydroxy-4'- methoxy-5-prenyl isoflavanone,  Apigenin,  Liquitirigenin,  Medicarpin,  phaseollidin,  Cristacarpin,  Sandwicensin,  2,4,4'- trihydroxychalcone | ^1^H and ^13^C NMR | Twigs, leaves, stem bark, stem wood and flowers of *E. lysistemon*. | *E. coli* | [86] |
| Epigallocatechin-3-gallate | Not detailed | Leaves of *C. sinensis* | *Stenotrophomonas maltophilia* | [91] |

**Caption:** Column Chromatography (CC), Thin Layer Chromatography (TLC), High-Performance Liquid Chromatography (HPLC), Ultraviolet-Visible Spectroscopy (UV-Vis), Nuclear Magnetic Resonance (NMR), Mass Spectrometry (MS), one- and two-dimensional NMR techniques and mass spectrometry (HR-ESIMS), High-Resolution Mass Spectrometry (HRMS), High-Resolution Electrospray Ionization Time-of-Flight Mass Spectrometry (HR-ESI-TOF-MS), Infrared Spectroscopy (IR), Gas Chromatography-Mass Spectrometry (GC-MS), Nuclear Magnetic Resonance (NMR), Fast Atom Bombardment (FAB) Electrospray Ionization (ESI), Electron Ionization Mass Spectrometry (EIMS), Liquid Chromatography coupled to Mass Spectrometry (LC-MS), 1H Nuclear Magnetic Resonance Spectroscopy (1H-NMR), Carbon Nuclear Magnetic Resonance Spectroscopy (13C-NMR), Attached Proton Test (APT), Mass Spectrometry (MS), Diode Array Detection (DAD), Circular Dichroism (CD), Ultra-High-Performance Liquid Chromatography-Mass Spectrometry (UHPLC-MS), Fourier Transform Infrared Spectroscopy (FT-IR), Vacuum liquid chromatography (VLC), Correlation Spectroscopy (COSY), Heteronuclear Multiple Bond Correlation (HMC), Distortionless Enhancement by Polarization Transfer - Quadrature (DEPTQ), Distortionless Enhancement by Polarization Transfer (DEPT), Heteronuclear Single Quantum Coherence (HSQC), Heteronuclear Multiple Bond Correlation (HMBC), Heteronuclear Multiple Quantum Coherence (HMQC).
