# Supplementary material for: Flavonoids: A Review of Antibacterial Activity Against Gram-Negative Bacteria
Source: Int J Microbiol. 2025 Sep 15;2025:9961121. doi: 10.1155/ijm/9961121 (PMC12453933; doi:10.1155/ijm/9961121)
Supplement: Supporting Information — Additional supporting information can be found online in the Supporting Information section. Table S1. Antibacterial activity of flavonoids isolated from plants against Gram-negative bacteria (2004–2025). [file 9961121.f1.zip › Response to the reviewer review.docx]

Response to the reviewer:

2.3 antibacterial activity of flavones: this section was found to be too lengthy and elaboratives. It is suggested for this section to trimmed down and highlighting only the essential part of flavones

**Response:** As suggested, the section on the antibacterial activity of flavones has been revised and simplified, with a focus on clarity and objectivity. Essential information has been retained in order to preserve the integrity and relevance of the data presented.

2.4 antibacterial activity of flavonols: this section was found to be too lengthy and elaboratives. It is suggested for this section to trimmed down and highlighting only the essential part of flavonols

**Response:** As suggested, the section on the antibacterial activity of flavonols has been revised and simplified, prioritizing clarity and objectivity. The essential information was retained to ensure the integrity and relevance of the data presented.

3. Mechanisms of actions of flavonoids : it is suggested to add some schematic diagrams, equation, flow chart or as such to support the explanation on how flavanoids acted as antibacterial/antimicrobial agents in the biological system.

**Response:** We appreciate the suggestion regarding the inclusion of diagrams, flowcharts, or equations to illustrate the mechanisms of action of flavonoids. However, we opted not to include such elements at this time due to the limited availability of specific mechanistic data in the reviewed articles, particularly concerning the detailed action of flavonoids against Gram-negative bacteria. Furthermore, the primary focus of this work was the compilation of experimental data on antibacterial activity. A graphical or schematic approach to the mechanisms would require a more in-depth and specific analysis, which goes beyond the scope of this review. Nonetheless, the antibacterial actions of flavonoids have been described textually, based on the available evidence.

References: to add more recent and up-to-date source of references. It is recommended to have 30-50% of the references were sourced from the latest publication (2020-2025). The list of publications from the document was found to be quite old and out of date (2004 and earlier).

**Response:** This review aimed to compile and analyze data on the antibacterial activity of flavonoids isolated from different plant species against Gram-negative bacteria, based on publications available from 2004 to 2025, accessed through various scientific databases. However, a scarcity of recent studies specifically focused on this topic was identified, which justified the inclusion of older references. Nevertheless, we have made efforts to prioritize, whenever possible, more recent and relevant studies to support the development of this work.

Additional Comments:

1. Please upload editable versions of the high resolution figures under Figure file section. None of the figures can be edited. We need to be able to edit lines, arrowheads, and fonts to match the journal’s style. Each figure should be a separate Word, Excel, PowerPoint or PDF file which can be edited. Please note that jpg, bmp, png, and tif files cannot be edited by default.

**Response:** The figures were created using ChemDraw Ultra and can be edited directly within the manuscript file. Additionally, the image is being submitted separately as an editable DOC file, as requested, allowing modifications to elements such as lines, arrows, and fonts in accordance with the journal’s style using the ChemDraw Ultra software.

2. Please upload a Clean Manuscript (yellow colour highlights) in DOC format . Please update the same on the Final Manuscript (in Docx format or PDF with TEX format) Please upload it under the main manuscript files section.

**Response:** As requested, all yellow highlights have been removed, and the clean version of the manuscript has been updated accordingly.
